# Impact of Supersonic Flow in Scintillator Detector Apertures on the Resulting Pumping Effect of the Vacuum Chambers

**DOI:** 10.3390/s23104861

**Published:** 2023-05-18

**Authors:** Jiří Maxa, Vilém Neděla, Pavla Šabacká, Tomáš Binar

**Affiliations:** 1 Institute of Scientific Instruments of the CAS, Královopolská 147, 612 64 Brno, Czech Republic; maxa@vutbr.cz; 2 Faculty of Electrical Engineering and Communication, Brno University of Technology, Technická 10, 616 00 Brno, Czech Republic

**Keywords:** Ansys Fluent, ESEM, scintillation detector, critical flow, one-dimensional flow theory, aperture, pressure sensor

## Abstract

The article describes the combination of experimental measurements with mathematical–physics analyses in flow investigation in the chambers of the scintillator detector, which is a part of the environmental scanning electron microscope. The chambers are divided with apertures by small openings that keep the desirable pressure differences between three chambers: The specimen chamber, the differentially pumped intermediate chamber, and the scintillator chamber. There are conflicting demands on these apertures. On the one hand, the diameter of the apertures must be as big as possible so that they incur minimal losses of the passing secondary electrons. On the other hand, it is possible to magnify the apertures only to a certain extent so the rotary and turbomolecular vacuum pump can maintain the required operating pressures in separate chambers. The article describes the combination of experimental measurement using an absolute pressure sensor and mathematical physics analysis to map all the specifics of the emerging critical supersonic flow in apertures between the chambers. Based on the experiments and their tuned analyses, the most effective variant of combining the sizes of each aperture concerning different operating pressures in the detector is determined. The situation is made more difficult by the described fact that each aperture separates a different pressure gradient, so the gas flow through each aperture has its own characteristics with a different type of critical flow, and they influence each other, thereby influencing the final passage of secondary electrons detected by the scintillator and thus affecting the resulting displayed image.

## 1. Introduction

Currently, the Institute of Instrumentation Technology of the Academy of Sciences of the Czech Republic in Brno is conducting research on environmental electron microscopy.

Environmental scanning electron microscope (ESEM) enables analyzing not only specimens under vacuum conditions, as does a more common scanning electron microscope (SEM), but also under various gasses with a pressure of thousands of Pa in the specimen chamber [1], which extends environmental scanning electron microscope’s application possibilities significantly [2]. The presence of higher-pressure gas within ESEM enables a specimen analysis [3] as well as displaying not only electrically non-conductive samples without plating them with metal even at higher electron-beam energies [4,5] but also damp biology specimens [6] or polymer specimens [7] under the condition of thermodynamic equilibrium without any desiccation nor damage. Another advantage of ESEM is the option of preparing the in-situ specimens and observing the unaltered surface directly [8] or studying specimen behavior under dynamically varying conditions [9]. Signal electrons in ESEM are detected by either special ionizing [10,11] or scintillating detectors [12,13], the image of which can correlate with one of a light microscope [14].

Generally, an ESEM consists of neighboring chambers separated by small apertures, which procure great pressure gradients between the chambers [10,11]. These considerable pressure gradients cause a critical gas flow, including supersonic flow with great pressure gradients [15,16,17]. Another possible part of the ESEM is a scintillation detector, which contains resembling chambers separated by apertures [12]. Topics presented in this article will be experimental measurements in correlation and mathematical physics analyses regarding flow in scintillation detector chambers, establishing the optimal condition for its operation. ESEM is specific by vacuum chambers separated by small apertures, in which supersonic flow is generated at very low pressures at the lower limit of continuum mechanics. The flow is very specific [18,19,20,21].

## 2. Theory of Critical Flow

A characteristic sign of the ESEM chambers is chambers separated by small apertures with great pressure gradients. A flow in such apertures separating chambers with significant pressure differences has peculiar physics properties and goes by the term of critical flow [22,23,24]. When the pressure gradients on both sides of an aperture differ, a flow going from the place with the higher pressure to the one with the lower pressure occurs. The flow velocity in the aperture increases, as does the pressure gradient difference ratio. This is true only until the velocity reaches the figure of 1 Mach, which is when the mentioned critical flow takes place [25,26]. Once the critical flow is reached, a flow velocity exceeding 1 Mach cannot be achieved by additionally increasing the pressure difference. The very same applies to mass flow over time, meaning that under no circumstances will the amount of gas passing through the aperture at 1 Mach be surpassed. With another increase in pressure difference, a supersonic flow occurs behind the aperture, in the area of lower pressure, since matter cannot pass anymore due to the critical flow [27,28].

**Figure 1:**
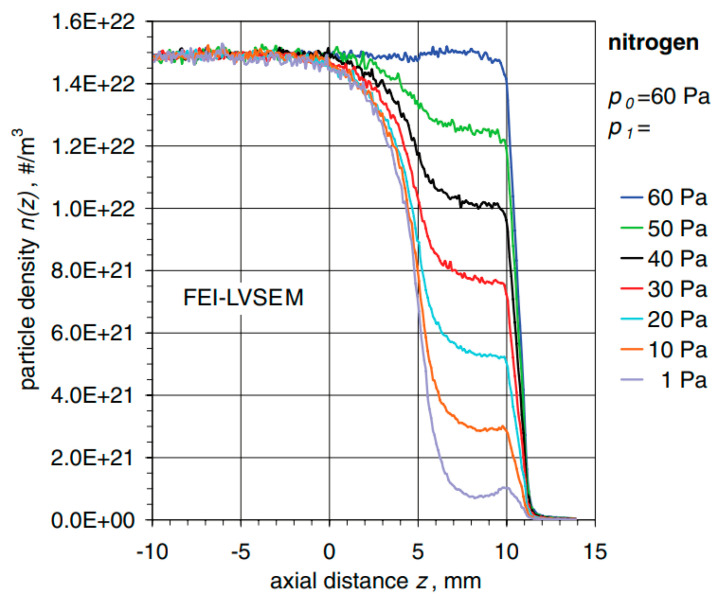
Shift in particle density regarding gaseous nitrogen at the pressure of 60 Pa [29].

**Figure 2:**
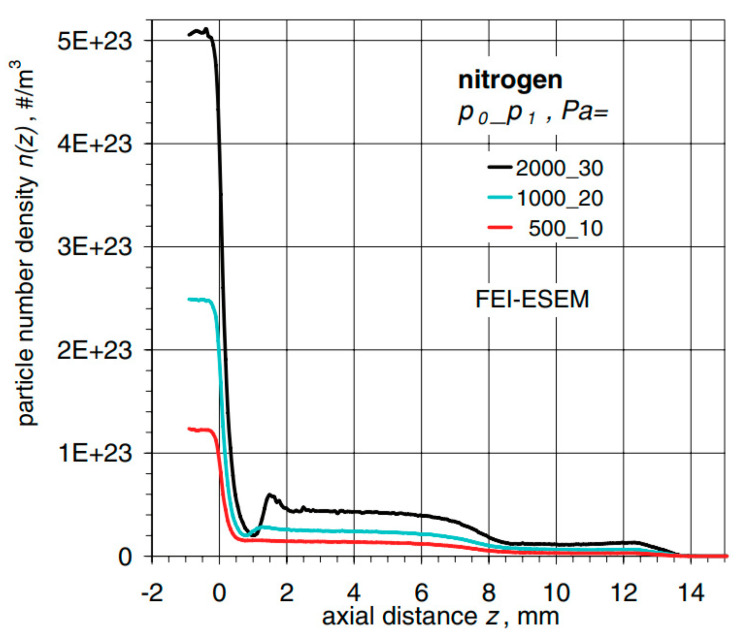
Shift in particle density [29].

Dr. Danilatos, an innovator of ESEM, paid closer attention to this problem in one of his articles, where he analyses gas flow through the aperture depending on its size and controlled back pressure. This can be seen in Figure 1 and Figure 2, taken from his publication, where FEI-LVSEM stands for Low Vacuum Scanning Electron Microscopy of the FEI company, and FEI-ESEM stands for the classic type of Environmental Scanning Electron Microscope of FEI company, where a pressure of up to 10,000 Pa can be maintained in the specimen chamber due to a differentially pumped chamber [29].

In Figure 1, the shift in particle density alongside the axial distance regarding the gaseous nitrogen flowing from the chamber with a sample drawn at 60 Pa to the drawn outlet kept at constant pressures shown for every curve is depicted [29].

Shift in particle density alongside an axial distance regarding gaseous nitrogen flowing from the specimen chamber maintained at pressure *p*_0_ and back pressure *p*_1_ depicted for every curve can be seen in Figure 2.

### 2.1. Theory of One-Dimensional Isoentropic Flow

The critical flow state arises in the aperture separating two chambers with a significant pressure gradient resulting in a flow velocity of 1 Mach or greater. Afterward, dependences of state quantities on one another, such as pressure, temperature, density, velocity, and Mach number, originate. The correlation of these state quantities is described by the theory of one-dimensional isentropic flow.

The aforementioned state quantities and their mutual relations are represented in the following equations (Equations (1)–(6)) [30].

(1) $$\frac{v_{v}}{v_{kr}}={\left[\frac{\left(\varkappa +1\right)M^{2}}{2+\left(\varkappa -1\right)M^{2}}\right]}^{\frac{1}{2}},$$

(2) $$\frac{v_{v}}{v_{o}}={\left[\frac{2}{2+\left(\varkappa -1\right)M^{2}}\right]}^{\frac{1}{2}},$$

(3) $$\frac{T_{v}}{T_{o}}=\frac{2}{2+\left(\varkappa -1\right)M^{2}},$$

(4) $$\frac{p_{v}}{p_{o}}={\left[\frac{2}{2+\left(\varkappa -1\right)M^{2}}\right]}^{\frac{\varkappa }{\varkappa -1}},$$

(5) $$\frac{\rho_{v}}{\rho_{o}}={\left[\frac{2}{2+\left(\varkappa -1\right)M^{2}}\right]}^{\frac{1}{\varkappa -1}},$$

(6) $$\frac{\rho_{v}}{\rho_{kr}}=\frac{A_{kr}}{A}=M{\left[\frac{\varkappa +1}{2+\left(\varkappa -1\right)M^{2}}\right]}^{\frac{1}{2}\frac{\varkappa +1}{\varkappa -1}},$$

where *p*_0_ is the input pressure, *p_v_* is the output pressure, *T*_0_ is the input temperature, *T_v_* is the output temperature, *v*_0_ is the input velocity, *v_v_* is the output velocity, *v_kr_* is the critical velocity, *ρ*_0_ is the input density, *ρ_v_* is the output density, *M* is the Mach number, ϰ is the gas constant = 1.4, *A* is the computational cross-section, and *A_kr_* is the critical cross-section.

## 3. Scintillation Detector of an Environmental Scanning Electron Microscope AQUASEM II

**Figure 3:**
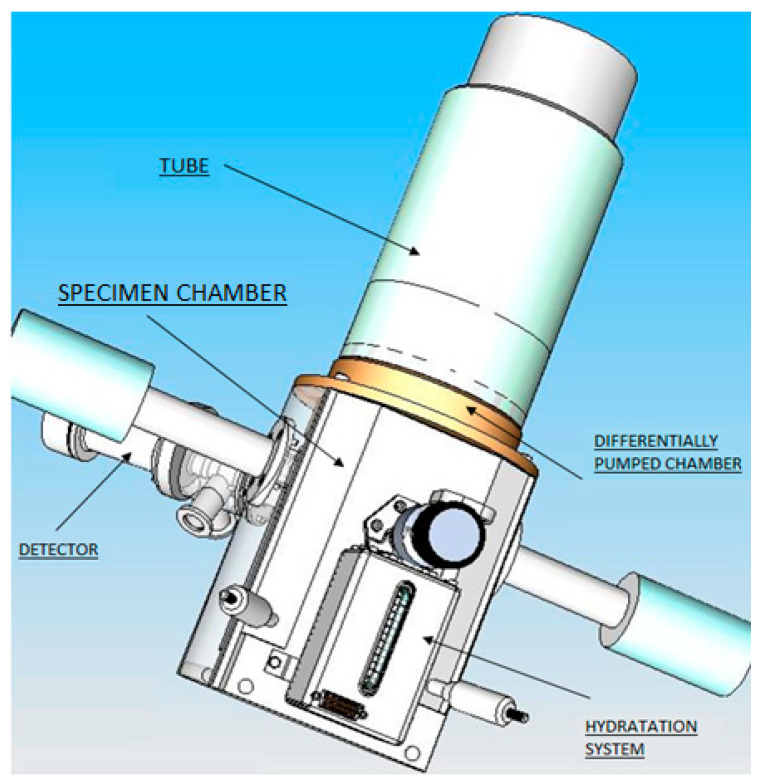
Electron microscope AQUASEM II—3D model.

**Figure 4:**
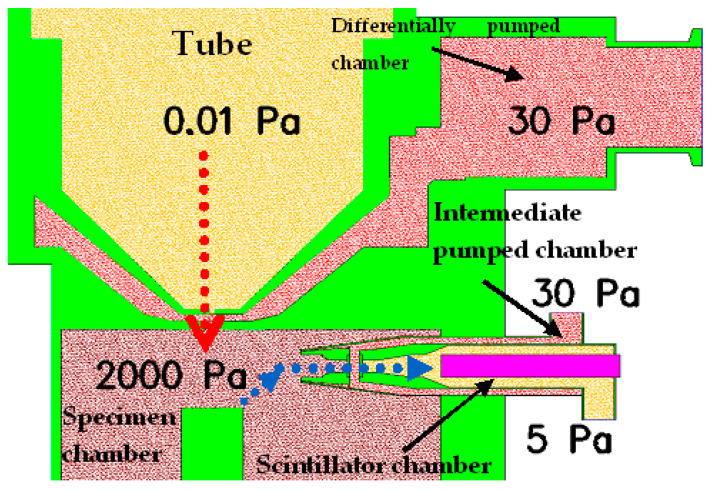
Electron microscope AQUASEM II—chamber diagram.

**Figure 5:**
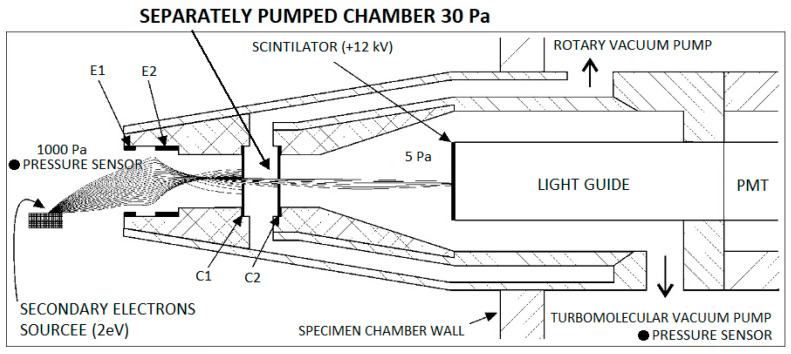
The principle of the scintillation secondary electron detector for ESEM with secondary electron with an energy of 2 eV path simulation (E1—extraction electrode, E2—deviation electrode, C1—aperture 1, C2—aperture 2, PMT—photomultiplier).

Several signals arise upon an electron batch hitting the surface of an examined specimen, such as secondary electrons (SE), backscattered electrons (BSE), transmitted electrons (TE), characteristics, and bremsstrahlung X-rays. We are able to obtain pieces of information about the structure, crystallography, morphology, and chemical composition of the specimen by evaluating these signals correctly. The addressed detector is a scintillation detector of secondary electrons for ESEM (Figure 3). The scintillation detector of secondary electrons is inserted into the specimen chamber from the side (Figure 4). Figure 3 shows a view of the overall ESEM showing a scintillation detector inserted from the side into the microscope. Figure 4 shows a schematic section guided given microscope and also inserted detector. The scintillation detector is further zoomed and described in Figure 5.

In the secondary electron scintillation detector in ESEM, the scintillator is placed in an individually drawn chamber separated by apertures C1 and C2 (Figure 5). It is an analogy of the differentially pumped chamber principle (Figure 4). Suitable potentials within an extent of a few hundred volts are attached to the apertures creating an electrostatic lens. Secondary electrons are routed to this lens by electrodes in the orifice of the detector and then pass to the scintillator. That enables us to connect up to 12 kV of voltage on the scintillator without causing discharges in the gas. High voltage on the scintillator speeds up electrons passing through the aperture with high enough energy to elicit scintillations. Photons created by the scintillations then traverse into a photomultiplier through a light guide, where they are amplified and transformed into an electric signal. The principle of a scintillation secondary electron detector is depicted in Figure 5. That enables us to connect up to 12 kV of voltage on the scintillator without causing discharges in the gas. High voltage on the scintillator speeds up electrons passing through the aperture with high enough energy to elicit scintillations. Photons created by the scintillations then traverse into a photomultiplier through a light guide, where they are amplified and transformed into an electric signal. The principle of a scintillation secondary electron detector is depicted in Figure 5.

## 4. Analysis of the Supersonic Flow Character in the Apertures of the Scintillation Detector

In the first step, Ansys Fluent system was tuned for performing mathematical physics analyses using results obtained from the experimental measuring on Environmental scanning electron microscope type AQUASEM II. The experimental measuring of pressure layout in operating conditions in the specimen chamber was performed using pressure sensors Pfeiffer CMR 361 of chosen pressure series: 1100, 1000, 900, 800, 700, 600, 500, 400, 300, 200, 100, 50 Pa. The measuring of the pressure layout in the scintillator chamber was performed using the pressure sensor Pfeiffer CMR 362. Both pressure sensor specifics are listed in Table 1. Using pressure sensors, the pressure in the specimen chamber and on the pumping throat of the scintillator in the detector was measured (Figure 5). It is necessary to mention that it was an operating variant of the detector with an aperture size of 0.6 mm in diameter.

**Figure 6:**
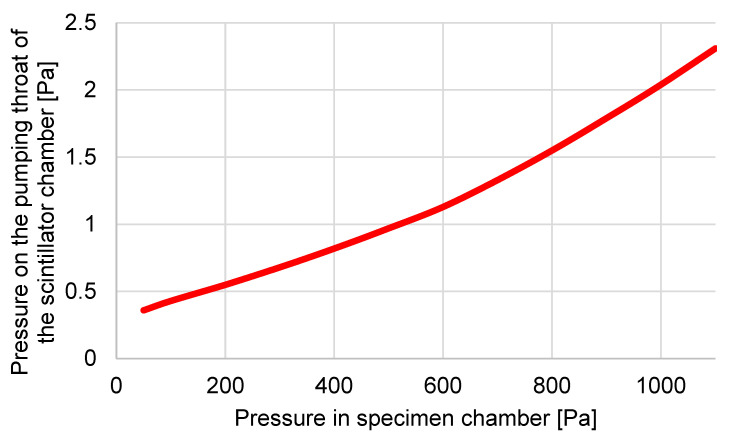
Results of experimental measuring of pressure capturing in ESEM AQUASEM II.

The results are listed in Table 2 and the graphic (Figure 6).

**Figure 7:**
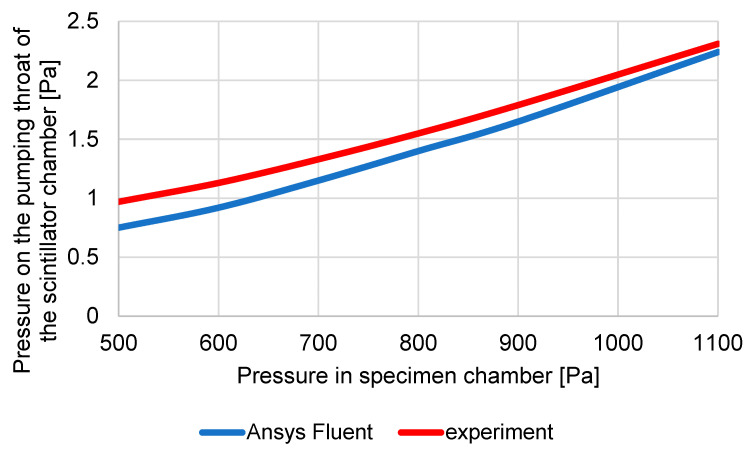
Comparison of results obtained by experimental measurements with results obtained by simulations in the Ansys fluent system.

From Table 1, it is clear that the differences in Figure 7, where the results of experimental measurements and analyses from the Ansys Fluent system are compared, are smaller than the error of sensors, which is max. 0.2 Pa.

At the same time, a 3D volume model of the detector was created, on which mathematical physics analyses were performed using the Ansys Fluent system and was tuned to correspond to the ESEM pumping conditions identical to the experimental measurement conditions. In this case, only selected pressures from the previous series were captured: 1100, 900, 800, 700, 600, and 500 Pa. The results and comparison of experimental measurements with the results of mathematical physics analyses are shown in Table 3 and in the graph (Figure 7).

The results show the corresponding compliance and appropriate tuning of the Ansys Fluent system. A suitable correspondence between experimental measurements using pressure sensors and mathematical physics analyses was demonstrated. However, the results of mathematical physics analyses were compared with the theoretical assumptions of supersonic flow in the nozzles. For this comparison, the above-mentioned theory of one-dimensional isoentropic flow was used (Equations (1)–(6)).

**Figure 8:**
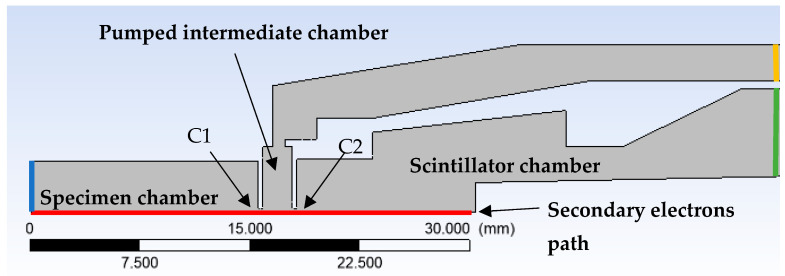
Cross-section of the front part of the detector showing the C1 and C2 apertures, specimen chamber, pumped intermediate chamber, and scintillator chamber together with the path of secondary electrons (red line), INPUT (blue line), OUTPUT 1 (green line), and OUTPUT 2 (yellow line).

Figure 8 shows the cross-section of the front part of the detector with C1 and C2 apertures, the front part of the detector extending into the specimen chamber, the pumped intermediate chamber, and the scintillator chamber. In Figure 8, there is a red path displayed on which the course of pressure quantities and velocity will be further plotted.

Subsequently, for a series of pressures: 1100, 900, 800, 700, 600, and 500, comparative analyses of mathematical physics analyses were performed using the Ansys Fluent system and the theory of one-dimensional isoentropic flow. In all cases, it is a variant with aperture diameters of 0.6 mm.

### 4.1. Variant of 1100 Pa in Specimen Chamber

**Figure 9:**
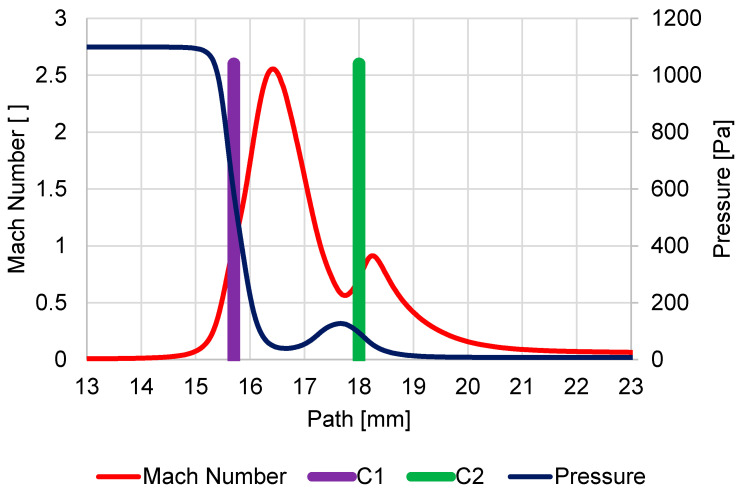
Course of Mach number and pressure on the secondary electron path under pressure conditions of 1100 Pa in specimen chamber.

In Figure 9, the course of Mach number and pressure on the secondary electrons path was displayed under pressure conditions of 1100 Pa in the specimen chamber, and the aperture size of the hole diameter C1 = 0.6 mm and C2 = 0.6 mm.

It is evident that due to the large pressure drop from 1100 Pa to 60 Pa on the first aperture C1, a supersonic critical flow reaching the Mach number value of 2.54 occurs. This leads to the so-called clogging of the nozzle, and the mass flow into the pumped intermediate chamber is limited, and it is clear from the pressure course that there is an area of reduced pressure behind the aperture C1. This area of reduced pressure in the supersonic flow area is terminated with a Mach disk.

Due to the nature of the smaller pressure gradient on the second aperture C2 60 Pa and 7.5 Pa, supersonic flow no longer occurs on this aperture, although the flow velocity reaches almost 0.9 Mach number. The pressure behind the aperture decreases uniformly without the Mach disk.

**Figure 10:**
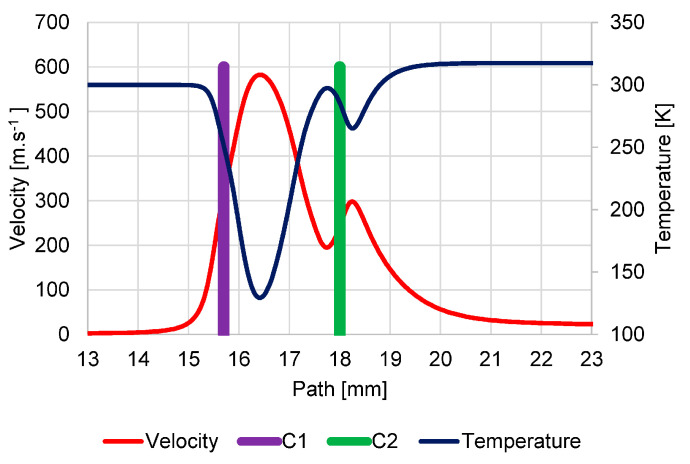
Course of velocity and temperature on the secondary electron path under pressure conditions of 1100 Pa in specimen chamber.

Supersonic flow is manifested by a characteristic decrease in temperature in the supersonic region, as shown in the graph (Figure 10), where temperature values drop up to 129.4 K. This graph also shows the flow velocity in units m·s^−1^, which by its nature copies the flow velocity in Mach number and is given because the Mach number is influenced by the environment in which it is evaluated. Therefore, to evaluate the results of mathematical physics analyses, it is necessary to state the value of the velocity in addition to the Mach number.

**Figure 11:**
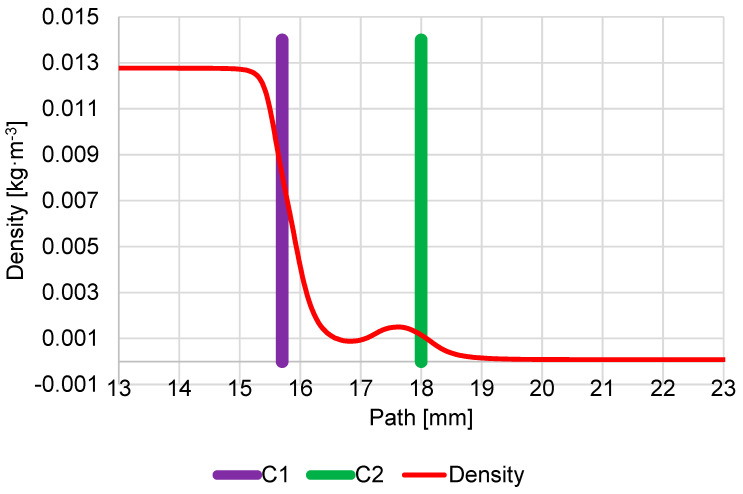
Course of density on secondary electron path under the pressure conditions of 1100 Pa in specimen chamber.

The density course looks similar to the pressure course (Figure 11).

All these quantities needed to be evaluated for subsequent comparison of the results of mathematical physics analysis obtained using the Ansys Fluent system with the theory of one-dimensional isoentropic flow presented in Section 2.1. This is because the flow physics in the nozzles, especially when the pressure gradient is reached, causing the critical flow mentioned in Section 2, closely relates to the state quantities of pressure, velocity, density, temperature, and the Mach number. If the results of mathematical physics analyses are consistent with the theory and supplemented by experimental measurements, we can say that the mathematical physics model is tuned and usable for analyses of aperture size changes without the need to produce all detector variants. In this methodology, the control experiment is usually used for the produced variant, which is determined as the most advantageous by mathematical physics analysis and as a verification of the correctness of tuning of mathematical physics analyses.

Table 4 shows the comparative results between the mathematical physics analyses and the theory of one-dimensional isoentropic flow, obtained from Equations (1)–(6), for the given pressure variant in specimen chamber 1100 Pa:

It can be noted that the results are in very good agreement. The correctness of the calculation is also confirmed by the flow velocity of 1 Mach directly in the aperture. Only the location of the Mach disk is slightly shifted to the aperture C1 because the character of the flow is influenced by the close location of aperture C2, which will be evident when comparing it with other variants where there is no longer such a large pressure gradient and the proximity of the aperture C2 is no longer so influencing, and the Mach disk will be localized in the calculation value.

**Figure 12:**
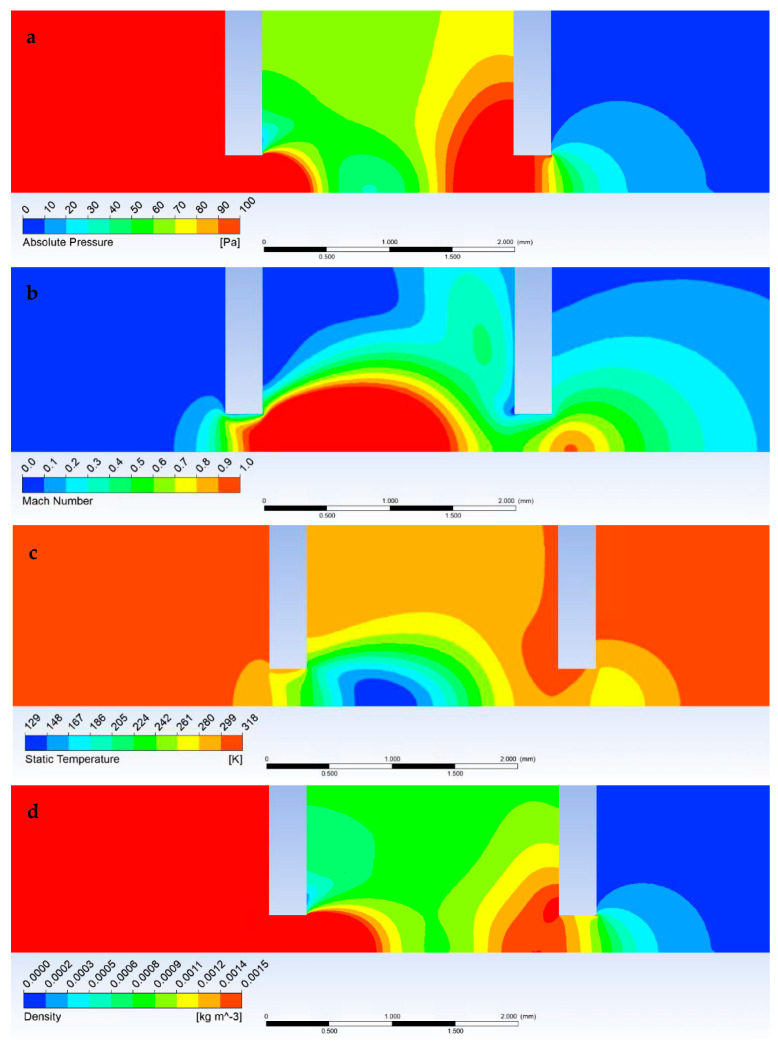
Graphical display of pressure (**a**), Mach number (**b**), temperature, (**c**) and density (**d**) in the specimen chamber, pumped inter-chamber, and scintillator chamber at pressure conditions of 1100 Pa in the specimen chamber.

Figure 12a shows a graphical representation of the pressure distribution shown in Figure 9. A distinct area of reduced pressure is visible, terminated by a Mach disk beginning at the end of the supersonic flow shown in Figure 12b. Similarly, in Figure 12c, the low-pressure area follows the distribution of the reduced temperature range. To complement and study the character of the flow, the density distribution in Figure 12d is given.

The given results fully correspond to the flow physics in the nozzles. The same analyses were carried out in the other selected pressures.

### 4.2. Variant 900 Pa in the Specimen Chamber

**Figure 13:**
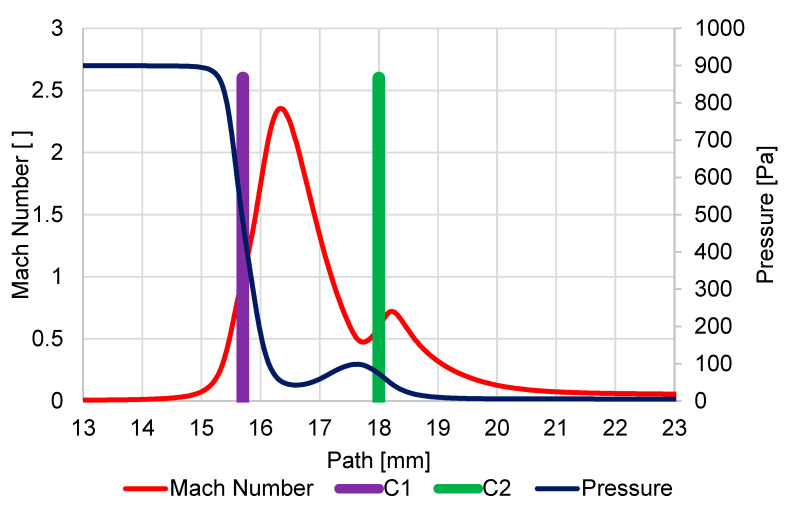
Course of Mach number and pressure on the path of secondary electrons under pressure conditions of 900 Pa in the specimen chamber.

In Figure 13, the course of Mach number and pressure on the secondary electron path is displayed under the pressure conditions of 900 Pa in the specimen chamber and apertures with the size of the hole diameter of C1 = 0.6 mm and C2 = 0.6 mm.

It is again evident that due to the still large pressure gradient of 900 Pa to 68 Pa on the first aperture C1, there is a supersonic critical flow logically reaching a slightly lower value of Mach number 2.34. Thanks to this, the so-called nozzle clogging occurs again, but with a slightly smaller consequence than in the previous case, which affects the pumping performance and results in slightly higher back pressure.

It is also noticeable that behind the aperture C1, there is an area of reduced pressure, but proportional to the magnitude of the supersonic flow. This area of reduced pressure in the supersonic flow area is terminated by a Mach disk.

On the second aperture C2, it is far from supersonic flow, and the pressure behind the aperture drops uniformly without a Mach disk.

**Figure 14:**
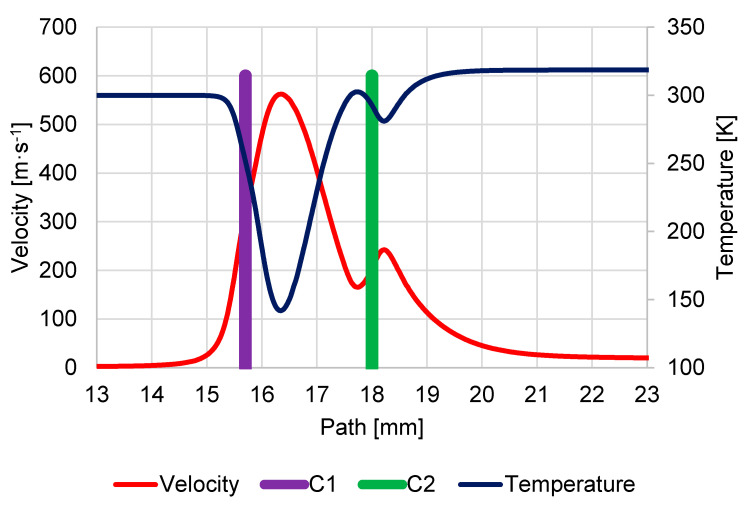
Course of velocity and temperature on secondary electron path under pressure conditions of 900 Pa in specimen chamber.

Moreover, the characteristic temperature drop in the supersonic region is slightly lower than in the previous case, as can be seen in the graph (Figure 14), where the temperature value drops to 142.1 K, and the flow velocity reaches 560 m·s^−1^.

**Figure 15:**
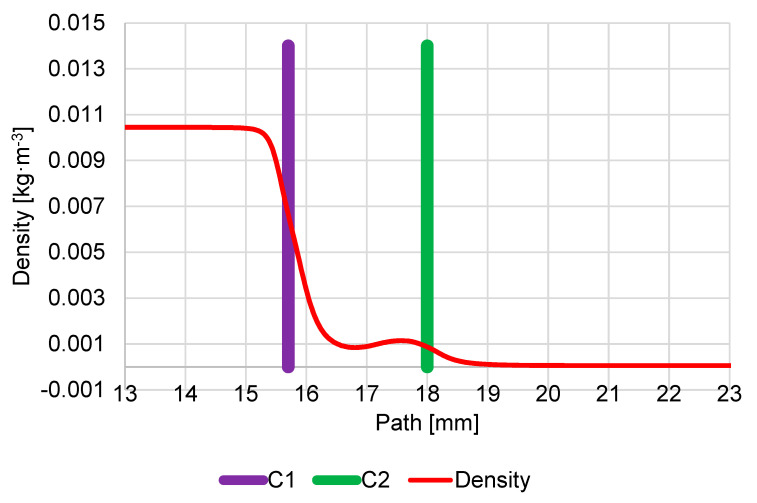
Course of density on the secondary electron path under pressure conditions of 900 Pa in specimen chamber.

The density course looks similar to the pressure course (Figure 15).

**Figure 16:**
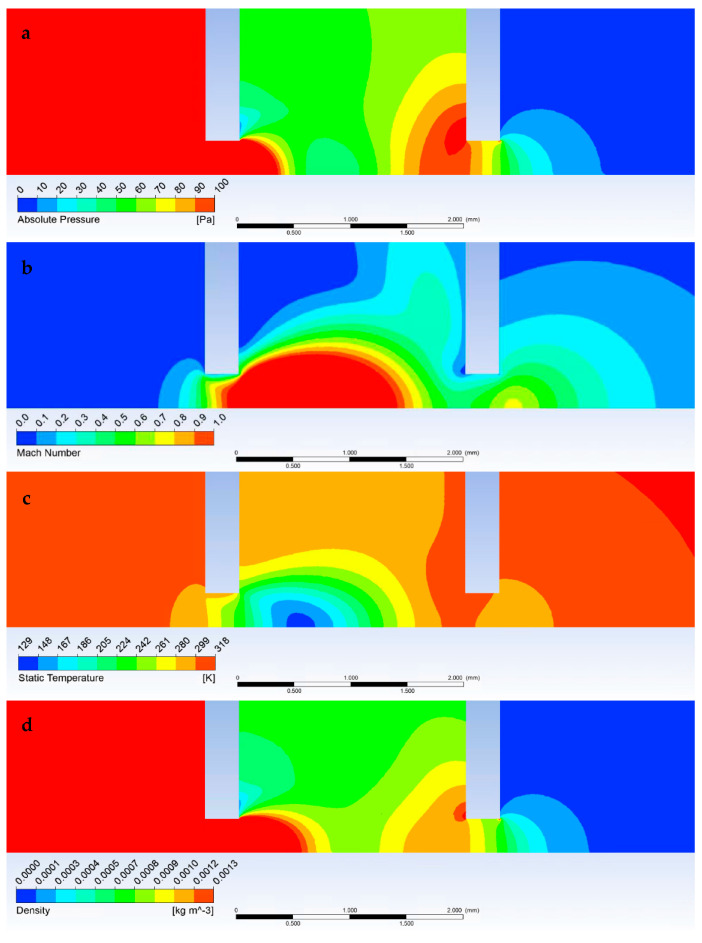
Graphical display of pressure (**a**), Mach number (**b**), temperature (**c**), and density (**d**) through the specimen chamber pumped inter-chamber and scintillator chamber at 900 Pa pressure conditions in the specimen chamber.

These results also correspond to the above-mentioned theory of one-dimensional isoentropic flow, obtained from Equations (1)–(6), and their mutual comparison is shown in Table 5:

In the following figures (Figure 16), it is possible to observe the characteristic distribution of the investigated quantities.

Other variants can be mentioned without further analysis, as it is similar to the previous variants and serves as a basis for a comparative analysis of the results presented here with the one-dimensional flow theory for checking the tuning of the Ansys Fluent system for the operating pressure range in ESEM. These variants with the brief description are shown in Appendix A (Appendix A.1, Appendix A.2, Appendix A.3 and Appendix A.4).

**Figure 17:**
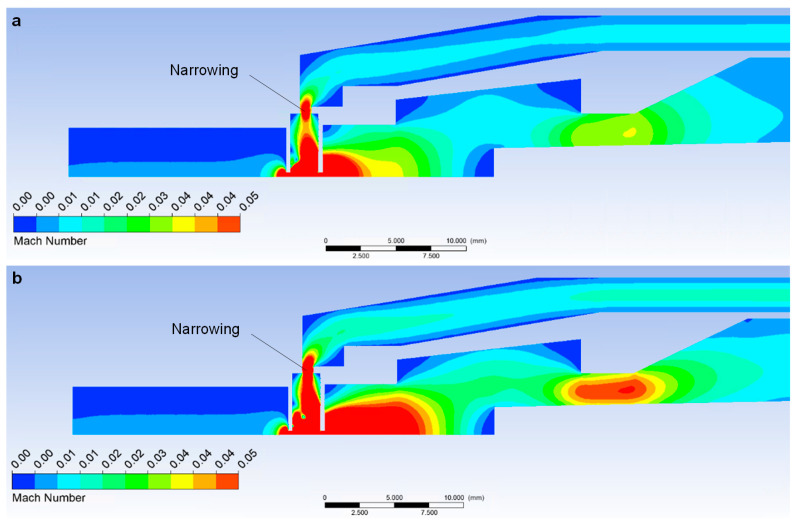
Distribution of Mach number in specimen chamber at pressure of 500 Pa (**a**) and 100 Pa (**b**).

One of the results of the mathematical physics analysis supported by experiment and theory is the finding that a small narrowing, seemingly irrelevant for pumping, causes an influence as if a third aperture on the pumping path of the scintillator chamber, as shown in Figure 17.

These figures show two examples of flow velocity in pumped channels.

In the upper figure (Figure 17a), it is a variant with a pressure of 500 Pa in the specimen chamber, in the lower one (Figure 17b), it is a variant of 1100 Pa.

**Figure 18:**
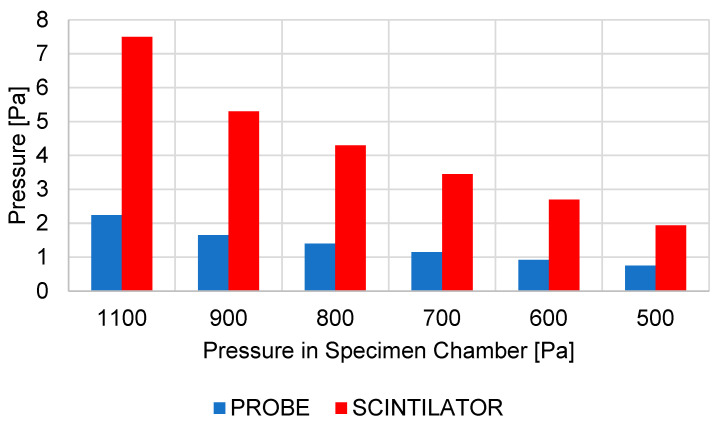
Graphical display of pressure comparison in specimen chamber.

In both cases, in the narrowing in the area of pumping of the scintillation chamber, there is an increase in the flow velocity in the narrowing (Figure 17) by up to 0.05 Mach number, even in the variant with half the pressure in the specimen chamber. This narrowing affects the quality of pumping of the scintillator chamber and causes a result that would not be solved by experimental measurements without a complex experiment. These are the increased pressure values of the scintillator, as shown in the graphical diagram in Figure 18.

Thanks to experimental measurements and analysis of the solved problem using supersonic flow theory, it was possible to appropriately tune the Ansys Fluent system to the specific type of flow that occurs during the pumping of ESEM chambers.

In the Ansys Fluent system, the Density Based solver was chosen as the equation solver due to the above-mentioned formation of supersonic flow with large pressure gradients. The advantage of this solver is that key equations, such as the equations of momentum, continuity, energy, and transport of substances, are solved simultaneously, which significantly contributes not only to the convergence of the calculation but, above all, the resulting values of temperature and flow velocity will respect the above-mentioned physical laws of mutual relations of state quantities in supersonic critical flow. Other equations are already solved sequentially. Two algorithms are available for solving a conjugate set of equations, the coupled-explicit formulation, and the coupled-implicit formulation. They differ in the way of linearization of conjugate equations. Of these two formulations, we chose the implicit formulation because of the complexity of the flow type. In the implicit formulation, each equation in the conjugate set of control equations is linearized implicitly concerning all dependent variables in the set [31].

Ansys Fluent solves the area of fluid dynamics using equations for the law of conservation of mass and momentum, in thermodynamics, also the law of conservation of energy [32,33,34]. The continuity equation formulates the law of conservation of mass in the field of fluid mechanics. For the elementary volume through which the fluid flows, the mass of the fluid must be constant, and, therefore, the total change in mass must be zero. Continuity equation in differential vector form for the transient spatial flow of compressible fluid:

(7) $$\frac{1}{\rho }\frac{D\rho }{Dt}+\nabla \vec{u}=0,$$

where *ρ* is the density of the liquid, *∇* is nabla operator, and $\vec{u}$ is fluid velocity vector.

Ansys Fluent uses the Navier–Stokes equations, which apply Newton’s second law of motion—the law of force. Navier–Stokes equations in component form:

(8) $$\frac{Du_{i}}{Dt}=\frac{\partial u_{i}}{\partial t}+u_{k}\frac{\partial u_{i}}{\partial x_{k}}=-\frac{1}{\rho }\frac{\partial p}{\partial x_{i}}+v\frac{\partial^{2}u_{i}}{\partial x_{k}\partial x_{k}},$$

The physical significance of the individual components listed in Equation (8):

$\frac{\partial u_{i}}{\partial t}$—Variability of the flow field in time
$u_{k}\frac{\partial u_{i}}{\partial x_{k}}$—Characterizes convection
$-\frac{1}{\rho }\frac{\partial p}{\partial x_{i}}$—Pressure gradient
$v\frac{\partial^{2}u_{i}}{\partial x_{k}\partial x_{k}}$—Effect of viscosity

The Navier–Stokes equations can also be expressed in vector form:

(9) $$\frac{D\vec{u}}{Dt}=\frac{\partial \vec{u}}{\partial t}+(\vec{u}\nabla )\vec{u}=-\frac{1}{\rho }\nabla p+v\nabla^{2}\vec{u}$$

where $\vec{u}$ is fluid velocity vector, *∇* is nabla operator, *ρ* is liquid density, *p* is modified pressure, and *v* is kinematic viscosity coefficient.

The modified pressure *p* is defined by Equation (10):

*p* = *P* + *ρΨ* (10)

where *P* is pressure, and *Ψ* is gravitational potential.

The kinematic viscosity coefficient *v* is defined as the ratio of dynamic viscosity *μ* and liquid density *ρ*:

(11) $$v=\frac{\mu }{\rho }$$

The energy equation is used to solve the system because the equation of state considers the internal energy of gases:

(12) $$\frac{\partial }{\partial t}\left(\rho E\right)+\nabla \left(\vec{u}\left(\rho E+p\right)\right)=\nabla \left(\lambda_{eff}\nabla T-\sum_{i}h_{i}{\vec{J}}_{i}+\left({\overset{=}{\tau }}_{eff}\vec{u}\right)\right)+S_{h}$$

where *ρ* is the density of the liquid, *E* is the total specific energy, *∇* is the nabla operator, $\vec{u}$ is the fluid velocity vector, *p* is static fluid pressure, *λ_eff_* is effective conductivity coefficient, *T* is thermodynamic temperature, *h_i_* is specific enthalpy of component *i*, ${\vec{J}}_{i}$ is diffusion component i, *τ_eff_* is effective friction tensor, and *S_h_* is Strouhal’s number.

The total specific energy *E* is defined as the sum of the internal and kinetic energies:

(13) $$E=U+\frac{1}{2}\vec{u}\vec{u}$$

The internal energy *U* is defined by:

(14) $$E=U+\frac{1}{2}\vec{u}\vec{u}$$

where *h* is enthalpy.

The effective conductivity coefficient *λ_eff_* is given by the sum of thermal *λ* and turbulent thermal *λ_t_* conductivity:

(15) $$\lambda_{eff}=\lambda +\lambda_{t}$$

Meaning of the components in Equation (12):

$\frac{\partial }{\partial t}\left(\rho E\right)$—Energy accumulation
$\nabla \left(\vec{u}\left(\rho E+p\right)\right)$—Inlet-outlet
$\lambda_{eff}\nabla T$—Conductivity component
$\sum_{i}h_{i}{\vec{J}}_{i}$—Energy diffusion
${\overset{=}{\tau }}_{eff}\vec{u}$—Friction
$S_{h}$—Volumetric source yield

A 3D volume model was created using Design Modeler as an axisymmetric model and, together with the boundary conditions, is marked in Figure 8.

INPUT—Detector throat entering the specimen chamber. Here, the value of the static pressure was set according to the solved variant of the pressure in the specimen chamber. This article works with the variants of pressure magnitude in the specimen chamber according to Table 2.

OUTPUT 1—pumping of the scintillator chamber. This chamber is pumped by a turbomolecular vacuum pump Pfeiffer TPD 011, at a pumping speed of 0.0044 m^3^·s^−1^. Accordingly, the pumping speed of 21.8 m·s^−1^ was set to the pumping throat cross-section with an area of 202 mm^2^.

OUTPUT 2—pumping of the intermediate chamber. This space is pumped by a Lavat RV 40/21 rotary vacuum pump at a pumping speed of 0.01 m^3^·s^−1^. Accordingly, the pumping speed of 49.5 m·s^−1^ was set to the pumping throat cross-section with an area of 202 mm^2^.

**Figure 19:**
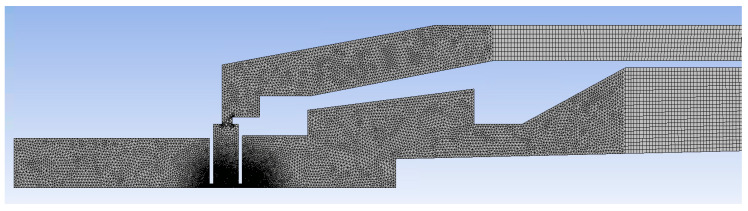
Mesh.

Mesh was created considering the expected values of pressure and velocity gradients. Especially in apertures, refining of up to 70 cells per cross-section and a sufficient number of cells on the cross-sections of the pumping channels was carried out. Mesh consists of a combination of tetrahedral and hexahedral cells, ensuring computational accuracy in pumping channels along with the possibility of saving the number of cells relative to their length. The form of the resulting mesh is in Figure 19.

Due to the very low Reynolds numbers due to low pressure, the laminar flow was set with the low-pressure boundary slip option, tested experimentally also in [23].

The given setting fully coped with this type of very complex flow and corresponded to the results of experimental measurements and the theory of one-dimensional isoentropic flow. It is a series of follow-up experiments and mathematical physics analyses gradually investigating conditions at the boundary of continuum mechanics for use in ESEM.

## 5. Analysis of the Impact of Apertures Pair Size on the Resulting Pumping Effect

Subsequently, using the tuned Ansys Fluent system, analyses of the effect of changing the size of the apertures pair on the resulting pumping effect were carried out.

### 5.1. Analysis of the Effect of Changes in Individual Apertures

In the first step, the effect of changing the size of the first or second aperture on the resulting effect of the ability of the vacuum pumps to maintain the required pressure parameters was investigated. Comparative analyses of the new variants with the original version were carried out, where the apertures have holes with a diameter of 0.6 mm, as mentioned in the previous chapter. The following versions in Table 6 were, therefore, compared.

**Figure 20:**
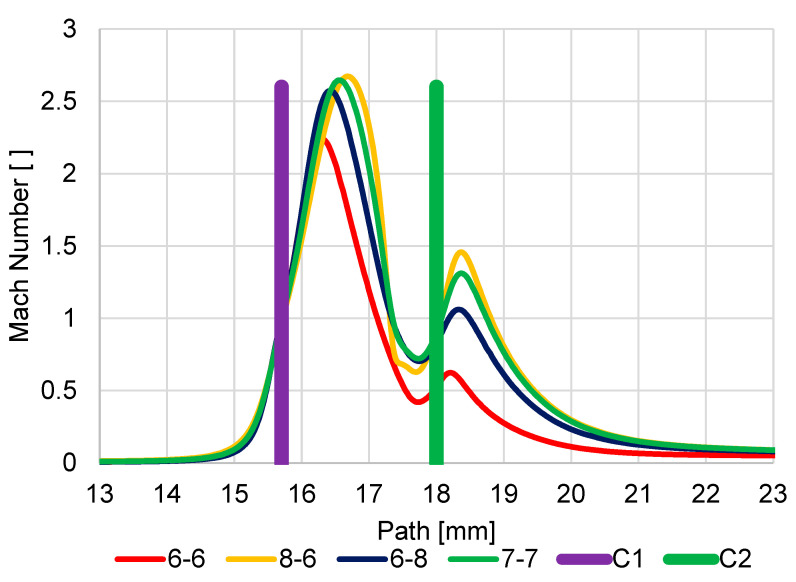
Dependence of aperture size on Mach number.

The first series of analyses showed that logically all new variants compared to the original version increase the value of the Mach number behind both apertures when the apertures are enlarged (Figure 20). However, it is important to analyze the new variants with regard to the impact of the change in dimensions for individual nozzles.

#### 5.1.1. Variant: Enlarged Second Aperture C2 (6-8)

**Figure 21:**
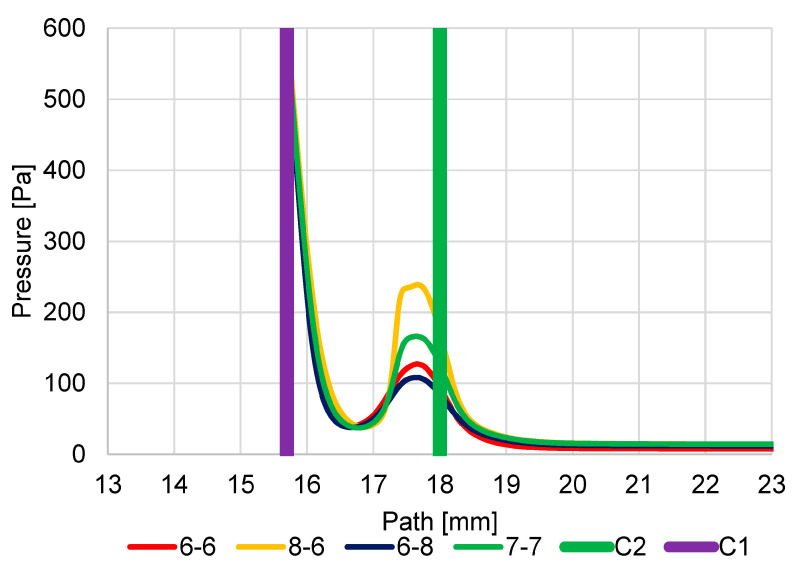
Dependence of aperture size on pressure course.

The Enlarged Second Aperture variant (6-8) has an increase in velocity slightly above 1 Mach and a greater mass flow rate on the enlarged second aperture (Figure 20). This results in less braking of the supersonic gas flow behind the first C1 aperture. The result of this change in velocities is a change in the pressure curve on the path (Figure 21). Because of the greater velocities behind the aperture C1 and, above all, the smaller decrease in velocity between the apertures and the re-increase in velocity above 1 Mach for aperture C2, there is less pressure increase at the end of the supersonic flow behind aperture C1. The average value of pressure on the path between the aperture C1 and the scintillator is, therefore, even lower in this variant than in the original variant, which is more favorable for detector operation.

#### 5.1.2. Variant: Enlarged First Aperture C1 (8-6)

In this variant, due to the enlargement of the first aperture, not only is there a noticeable increase in velocity but, above all, a significant elongation of the length of the supersonic flow area, so much so that the space defined by the two apertures is insufficient for this length. It is evident that this supersonic flow is already forcibly inhibited by the influence on the given velocity by the close location of the second aperture C2. This forced braking can also be seen when comparing the velocity distribution for the different variants in Figure 20. This forced braking results in a sharp increase in pressure at the end of the supersonic flow (Figure 21), which has the effect of artificially increasing the pressure gradient on the aperture C2 and so beyond this aperture C2, the flow velocity reaches almost 2 Mach. As a result, this variant proves to be very disadvantageous precisely because of the large average value of pressure on the path of secondary electrons.

#### 5.1.3. Variant: Equally Enlarged Both Apertures (7-7)

For comparison, an analysis was also carried out, where both apertures were evenly increased by 0.1 mm to a diameter of 0.7 mm compared to the original variant and compared with previous variants.

The result is a variant that corresponds to the average between the Enlarged Second Aperture variant (6-8) and the Enlarged First Aperture C1 variant (8-6). It does not bring any improvement over the enlarged second aperture C2 (6-8), but it does not have a problem with space as the Enlarged First aperture C1 variant (8-6).

**Figure A17:**
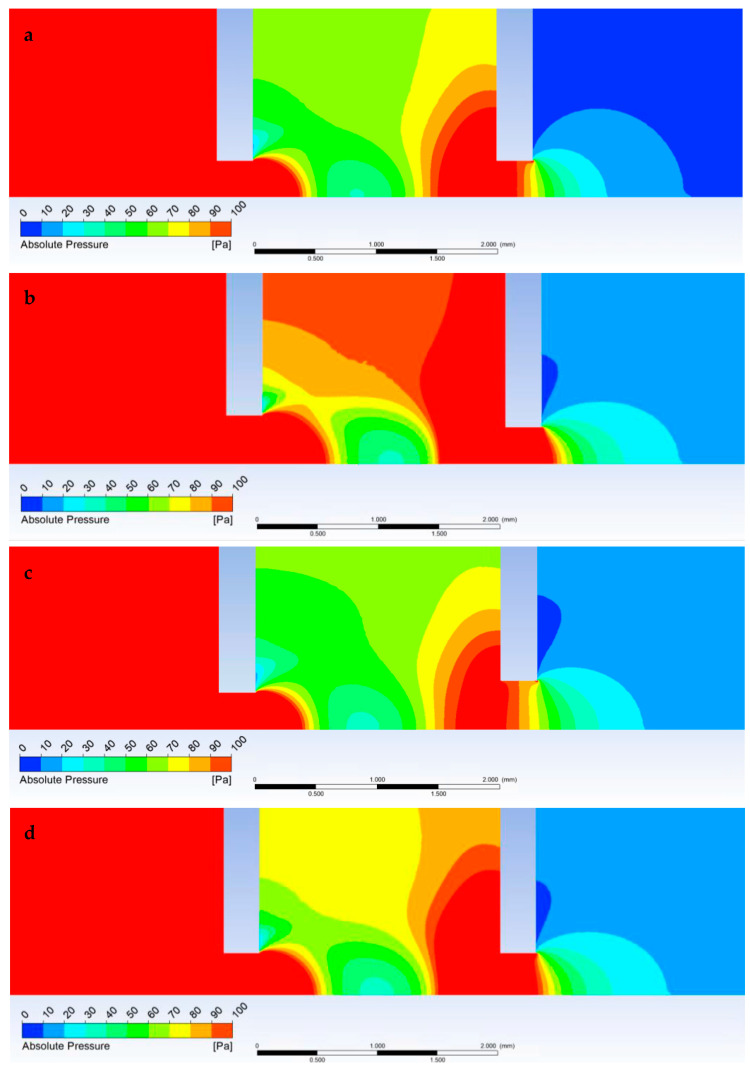
Pressure distribution for variants 6-6 (**a**), 8-6 (**b**), 6-8 (**c**), and 7-7 (**d**).

**Figure A18:**
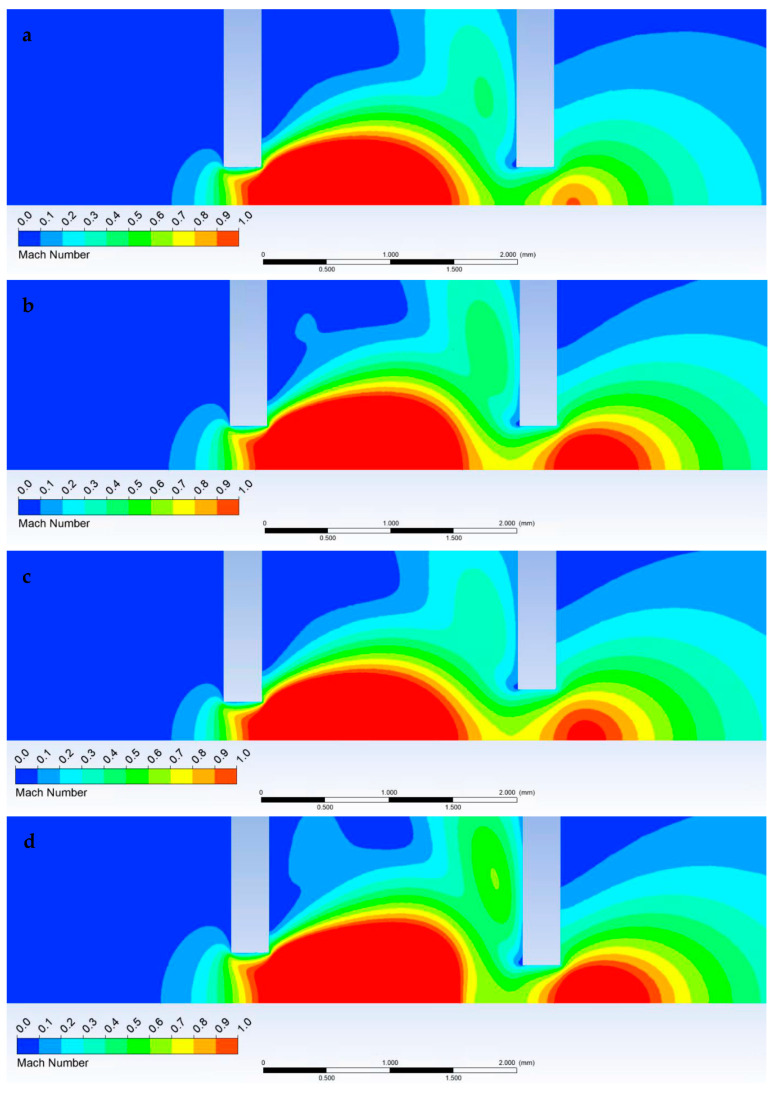
Mach number distribution for variants 6-6 (**a**), 8-6 (**b**), 6-8 (**c**), and 7-7 (**d**).

In Figure A17 and Figure A18 listed in Appendix A, the distribution of Mach Number and pressures are shown for a more detailed understanding of the results shown in the graphs (Figure 20 and Figure 21). A distortion of the Mach Number distribution in the 8-6 variant is evident (Figure A18b), which demonstrates the violent termination of the supersonic flow behind the aperture C1.

From this series of analyses, a subsequent series of analyses were carried out, dealing with a slight reduction in the diameter of the first aperture C1 to a diameter of 0.5 mm and a gradual increase in the size of the second aperture C2.

### 5.2. Analysis of the Effect of the Change in the Diameter of the Second Aperture C2

In the second step, the effect of changing the size of the second aperture on the resulting effect of the ability of the vacuum pumps to maintain the required pressure parameters was investigated. Comparative analyses of the selected variants were carried out, again with the original version, where the apertures have holes with a diameter of 0.6 mm, as mentioned in the previous chapter. The following versions in Table 7 were, therefore, compared.

**Figure 22:**
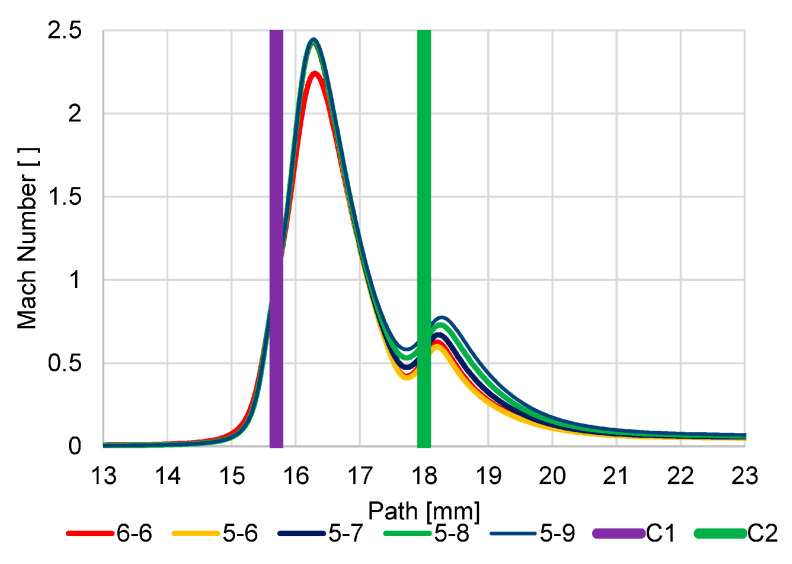
Dependence of aperture size on Mach number.

The second series of analyses showed the expected change that all new variants compared to the original version with the reduction of the first aperture C1 show an increase in the value of Mach number and increased expansion. In this case, it is due to the greater pressure gradient on the first aperture C1 caused by more efficient pumping to reduce the diameter of the aperture C1. However, due to the lower mass flow rate, the expansion returns to the subsonic velocity at the same distance beyond aperture C2 (Figure 22). Analyses of the new variants showed that the second gas expansion on the aperture C2 with respect to the dimension of C2 in individual apertures is proportional to the increase in the aperture C2 compared to the original variant. While maintaining the original dimension with a diameter of 0.6 mm, the Mach Number is almost identical to the original variant. Increasing the diameter of the aperture C2 has the effect of increasing the Mach Number on this aperture.

**Figure 23:**
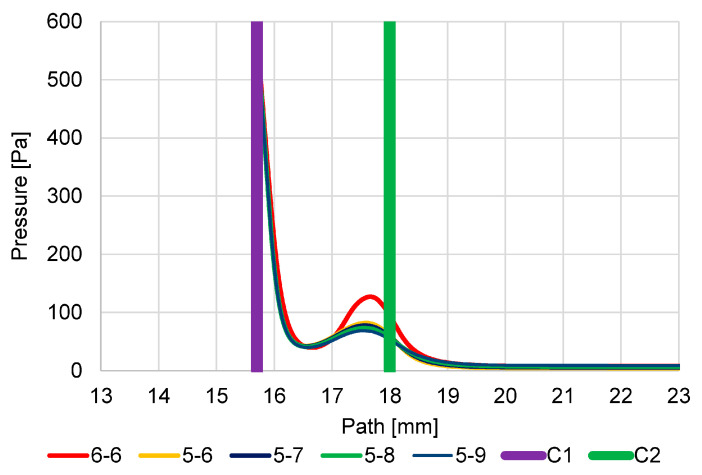
Dependence of aperture size on pressure course.

A change in flow velocity logically affects the course of pressure. In general, a reduction in the diameter of an aperture C1 causes, in all cases, a significantly lower pressure increase at the end of the supersonic flow beyond the first aperture C1 (Figure 23). Conversely, the larger the diameter of aperture C2, the lower the pressure increase at the end of the supersonic flow behind aperture C1. It is a difference of about 10 Pa between the two extreme values of the C2 average.

**Figure 24:**
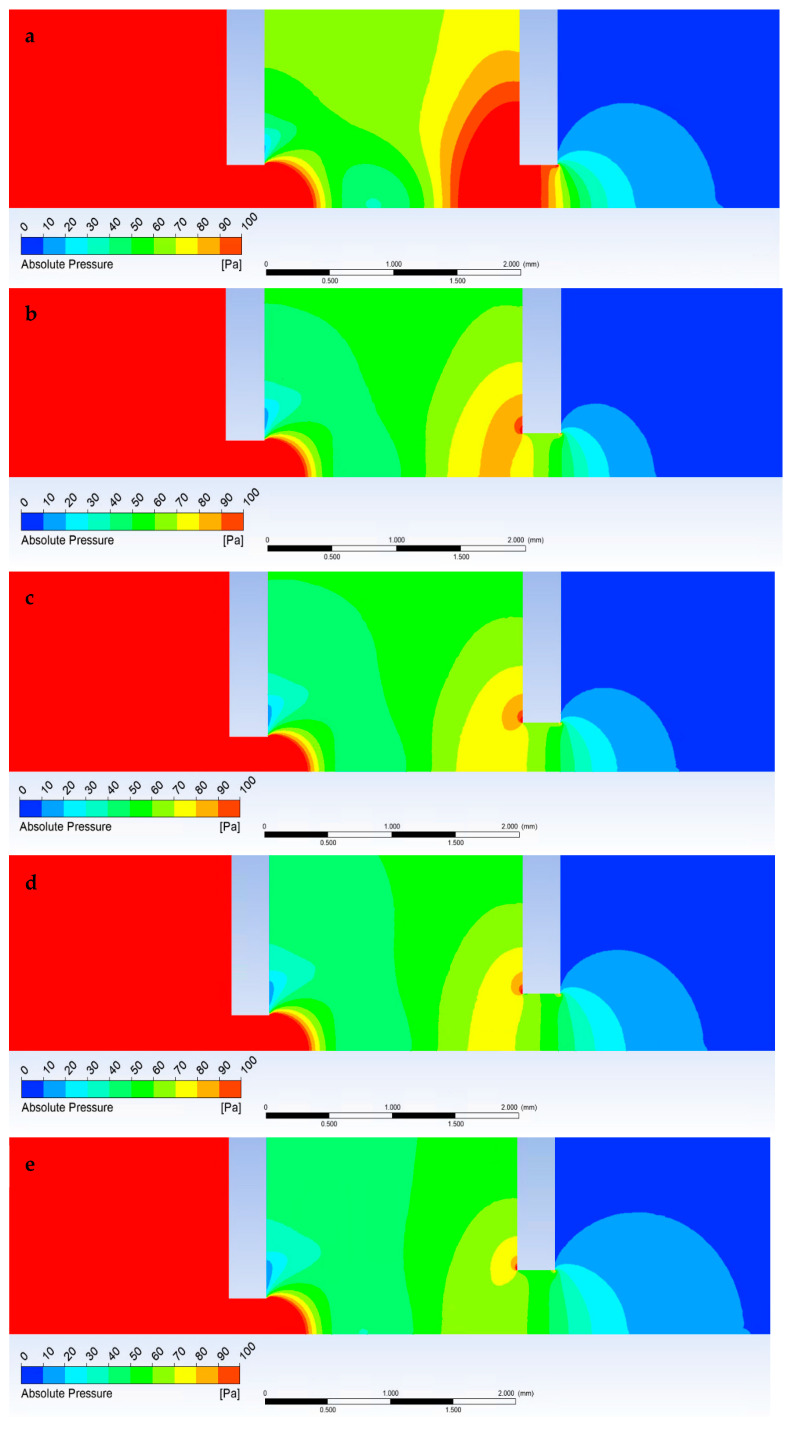
Pressure distribution for variants 6-6 (**a**), 5-6 (**b**), 5-7 (**c**), 5-8 (**d**), and 5-9 (**e**).

**Figure 25:**
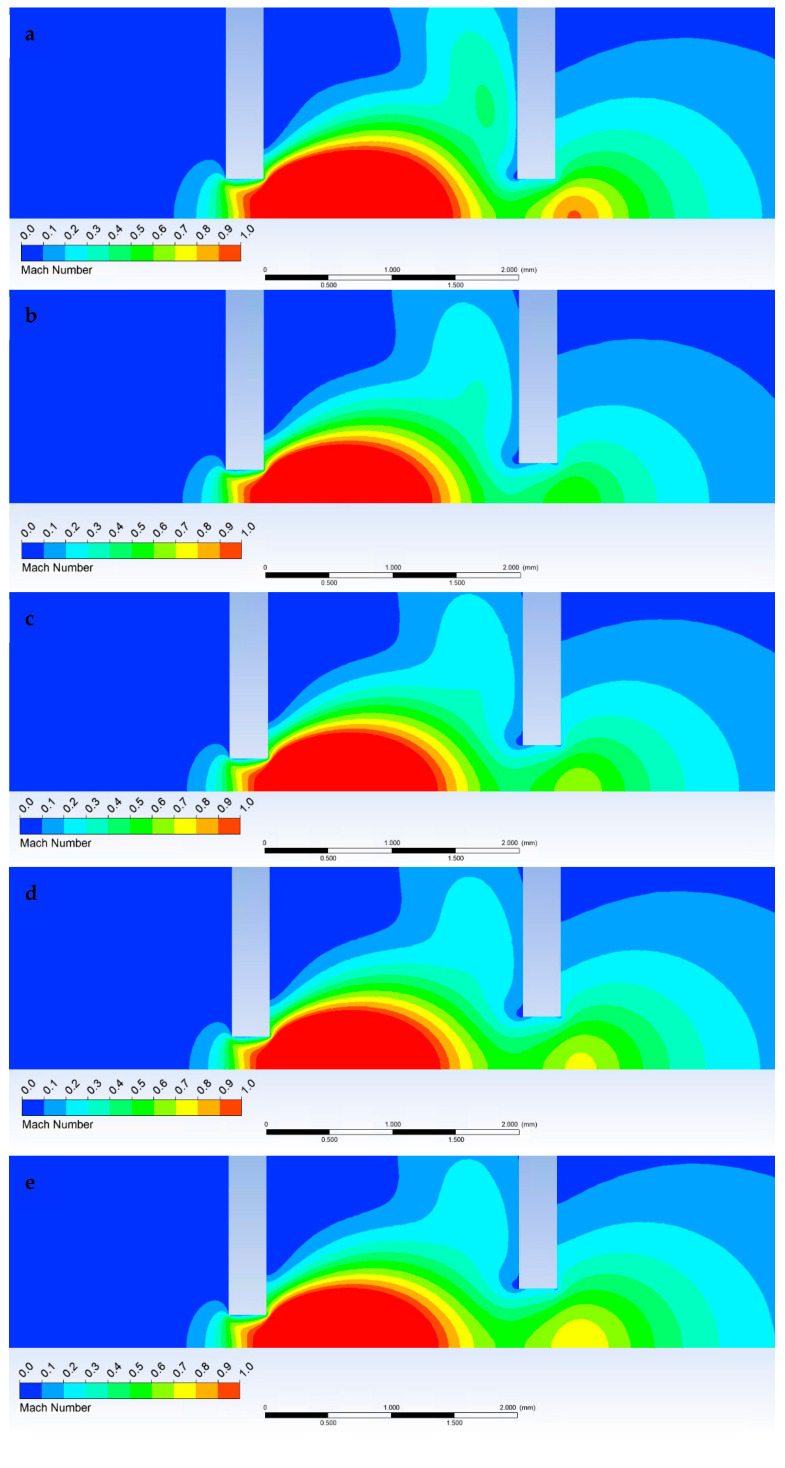
Mach number distribution for variants 6-6 (**a**), 5-6 (**b**), 5-7 (**c**), 5-8 (**d**), and 5-9 (**e**).

The following figures (Figure 24 and Figure 25) show the pressure distribution for a more detailed understanding of the results shown in the graphs (Figure 22 and Figure 23).

## 6. Results

**Figure 26:**
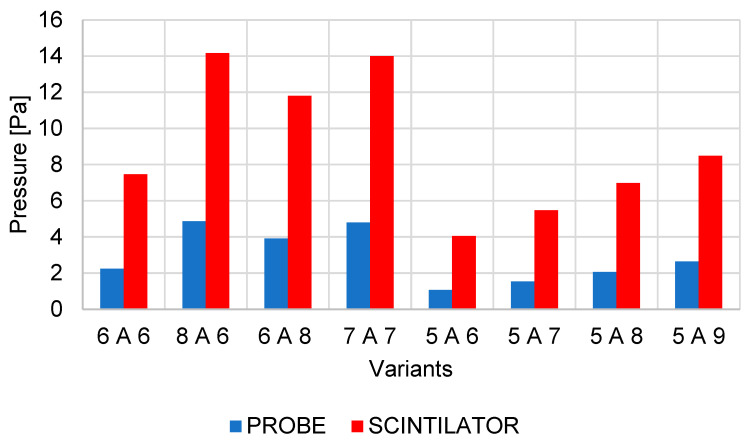
Graphical display compares pressures in specimen chamber.

The above conclusions describing the flow pattern in the detector explain the great impact on the operating pressure of the detector in the scintillator chamber. Figure 26 shows the summary results of pressures both from the sensor location at the pumping throat of the scintillator chamber and from the scintillator location (Figure 5). The results evaluate both selected locations due to the previously stated reason for a slight narrowing of the pumping channel (Figure 17).

The first four results relate to the first series of results, where the effect of changing the size of the first or second aperture on the resulting effect of the ability of the vacuum pumps to maintain these required pressure parameters was examined. The results copy the previously described effects of aperture sizes on the course of average gas pressure in the path behind the apertures C1 and C2. The highest pressure at the scintillator, as well as at the sensor location, is reached when the first aperture C1 is magnified. Conversely, the smallest pressure increase in this series of analyses is achieved when the second aperture C2 is enlarged. Taking into account that the 7-7 variant with a slight increase in both apertures is similar in value compared to the other two variants 8-6 and 6-8, it is clear that the impact of the size of the first aperture C1 on the resulting pressure of the scintillator is more significant.

The results of the second series of analyses evaluating the effect of the size of the second aperture C2 showed that even a slight reduction in aperture C1 has a very significant effect on the reduction of the resulting pressure in the scintillator and allows aperture C2 to increase (Figure 26). Variant 5-8 is comparable in its results to the original variant when increasing the sum of the two cross-sections of apertures C1 and C2. Variant 5-8 is, therefore, more advantageous for operating conditions than the original variant because it allows to achieve comparable thermodynamic conditions and, at the same time, increases the overall cross-section of the openings of both apertures for the passage of secondary electrons. If the operating pressure in the specimen chamber does not exceed 1100 Pa, it is theoretically possible to use variant 5-9, which is at the limit of the detector operating conditions, with a certain margin. If exceeded, the detector could be damaged by a discharge from the scintillator.

## 7. Conclusions

The paper deals with the problematics of pumping vacuum chambers at ESEM, which are separated by small apertures that provide large pressure gradients between individual chambers. These large pressure gradients cause critical gas flows containing supersonic flows with large pressure gradients. In this paper, experimental measurements were presented in cooperation with mathematical physics analyses analyzing the flow in the chambers of the scintillation detector, creating optimal conditions for its operation.

The effect of the change in the size of both apertures on the character of the supersonic flow and its effect on the resulting pressure in the scintillator chamber was analyzed.

The analyses were carried out in two series. In the first series, the impact of changing the sizes of both apertures was investigated, and the significant impact of the enlargement of the first aperture on the resulting impact of pumping the scintillator chamber was evaluated. The fundamental impact of the change in the diameter of the first aperture C1 on the resulting effect has been demonstrated.

In the second step, the effect of the size of the second aperture C2 at the same size as the first aperture C1 was evaluated in order to evaluate to what extent it is possible to enlarge the second aperture C2 to increase the intercepted cross-section while maintaining the operating conditions of the detector.

The results recommended to use variants 5-8 for detector operation, which is comparable in its results to the original variant, but compared to the original variant, has a significantly larger sum of both cross-sections of apertures C1 and C2, which will reduce the loss of passing secondary electrons that will not be captured by both apertures. With the certainty that the operating pressure in the specimen chamber over 1100 Pa is not exceeded, the results showed that, theoretically, it is possible to use variant 5-9, which is at the limit of the operating conditions of the detector.

The results show the corresponding agreement and suitably selected tuning of the Ansys Fluent system in the area of solver settings, discretization scheme, mesh, etc. A suitable correspondence between experimental measurements using pressure sensors and mathematical physics analysis was demonstrated. However, the results of mathematical physics analyses were also compared with the theoretical assumptions of supersonic flow in the nozzles. For comparison, the theory of one-dimensional isoentropic flow was used.

## Figures and Tables

**Table 1:** Specifics of pressure sensors Pfeiffer CMR 361 and Pfeiffer CMR 362.

	Pfeiffer CMR 361	Pfeiffer CMR 362
Measuring range [Pa]	10–11,000	10–110
Precision: % of measurement [%]	0.2	0.2
Pressure max. [hPa]	3000	2000
Response time [ms]	30	30

**Table 2:** Results of experimental measuring of pressure capturing in ESEM AQUASEM II.

Pressure in Specimen Chamber [Pa]	Pressure in Pumping Throat [Pa]
50	0.36
100	0.43
200	0.55
300	0.68
400	0.82
500	0.97
600	1.13
700	1.33
800	1.55
900	1.79
1000	2.04
1100	2.31

**Table 3:** Comparison of results obtained by experimental measurements with results obtained by simulations in the Ansys fluent system.

Pressure in Specimen Chamber [Pa]	Pressure in Pumping Throat [Pa]	Pressure in Pumping Throat Ansys [Pa]
500	0.97	0.75
600	1.13	0.92
700	1.33	1.15
800	1.55	1.4
900	1.79	1.65
1100	2.31	2.24

**Table 4:** Comparison of Ansys Fluent results with one-dimensional isoentropic flow theory.

	Ansys Fluent	One-Dimensional Flow Theory
P_0_ [Pa]	1100	1100
P_v_ [Pa]	60	60
Mach Number [-]	2.54	2.55
Velocity [m·s^−1^]	582	581
Temperature [K]	129.4	129.2
Density [kg·m^−3^]	0.0015	0.00161
Mach Disk [mm]	1.6	1.72

**Table 5:** Comparison of results obtained using Ansys Fluent system with the theory of one-dimensional isoentropic flow.

	Ansys Fluent	One-Dimensional Flow Theory
P_0_ [Pa]	900	900
P_v_ [Pa]	68	68
Mach Number [-]	2.34	2.34
Velocity [m·s^−1^]	560	558.6
Temperature [K]	142.1	141.8
Density [kg·m^−3^]	0.00114	0.00166
Mach Disk [mm]	1.49	1.46

**Table 6:** Variants and sizes of apertures for subsequent analyses.

	Further Marked	Diameter C1 [mm]	Diameter C2 [mm]
Original version	6-6	0.6	0.6
Enlarged first aperture	8-6	0.8	0.6
Enlarged second aperture	6-8	0.6	0.8
Uniform enlargement of both apertures	7-7	0.7	0.7

**Table 7:** Aperture variants and sizes for subsequent analyses.

	Further Marked	Diameter C1 [mm]	Diameter C2 [mm]
Original version	6-6	0.6	0.6
C2 = 0.6 mm	5-6	0.5	0.6
C2 = 0.7 mm	5-7	0.5	0.7
C2 = 0.8 mm	5-8	0.5	0.8
C2 = 0.9 mm	5-9	0.5	0.9

**Figure A1:**
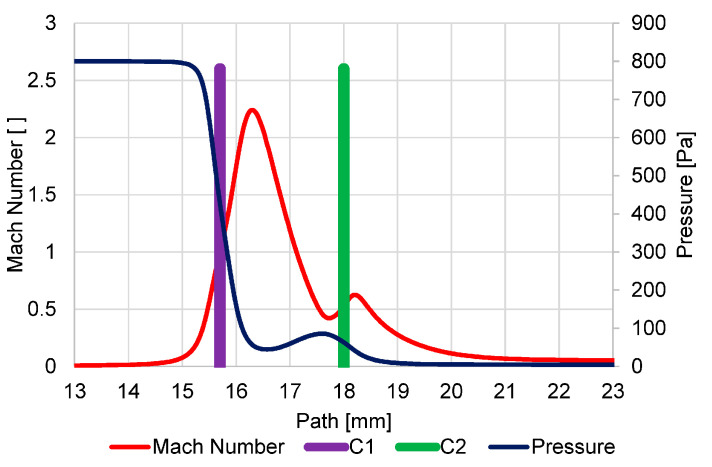
Course of Mach number and pressure on secondary electron path under pressure conditions of 800 Pa in specimen chamber.

**Figure A2:**
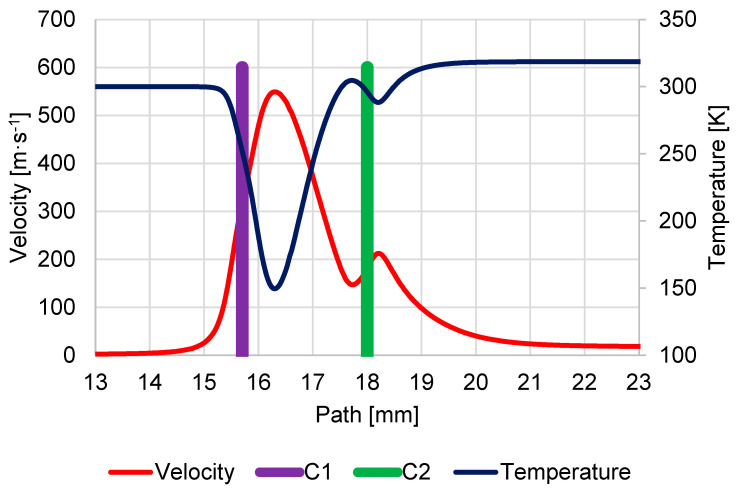
Course of velocity and temperature on secondary electron path under pressure conditions of 800 Pa in specimen chamber.

**Figure A3:**
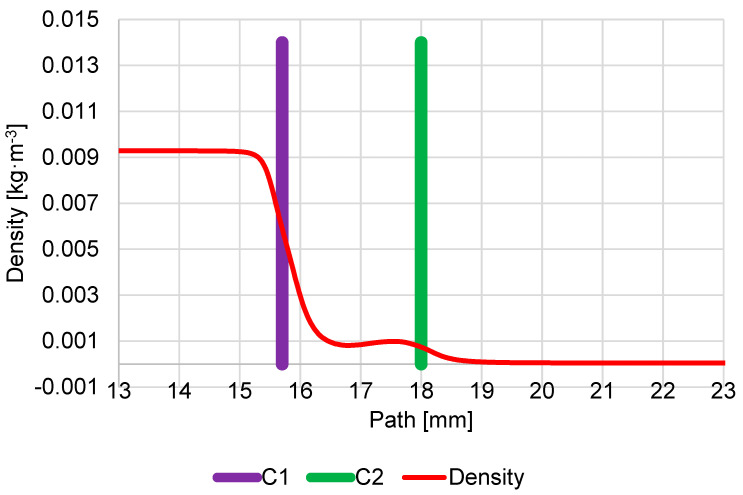
Course of density on secondary electron path under pressure conditions of 800 Pa in specimen chamber.

**Figure A4:**
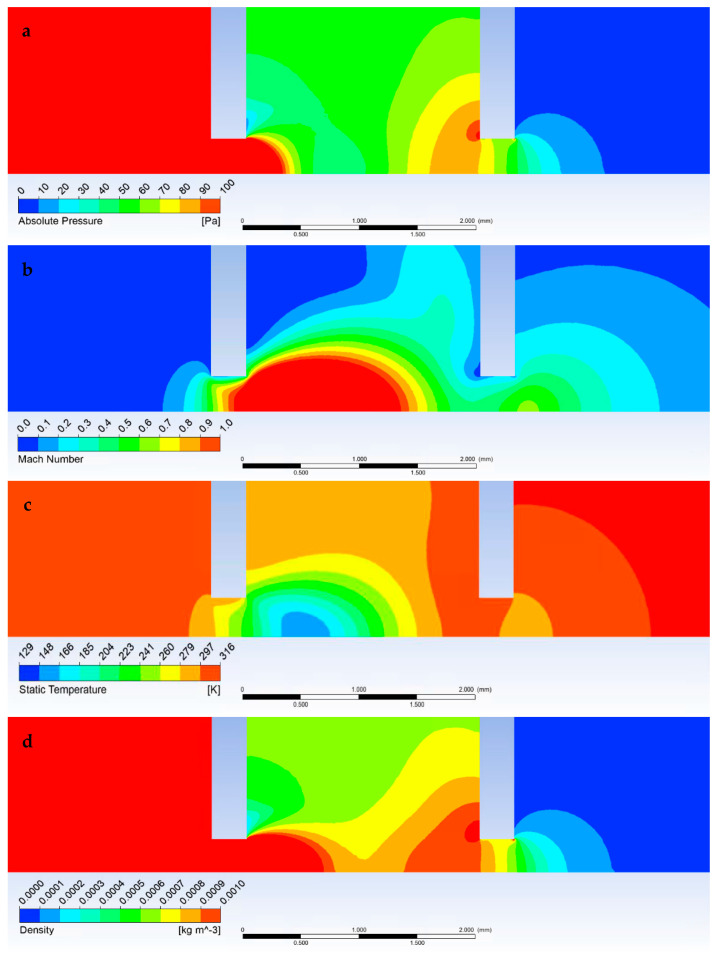
Graphical display of pressure (**a**), Mach number (**b**), temperature (**c**), and density (**d**) of the specimen chamber, pumped inter-chamber and scintillator chamber at 800 Pa pressure conditions in the specimen chamber.

**Figure A5:**
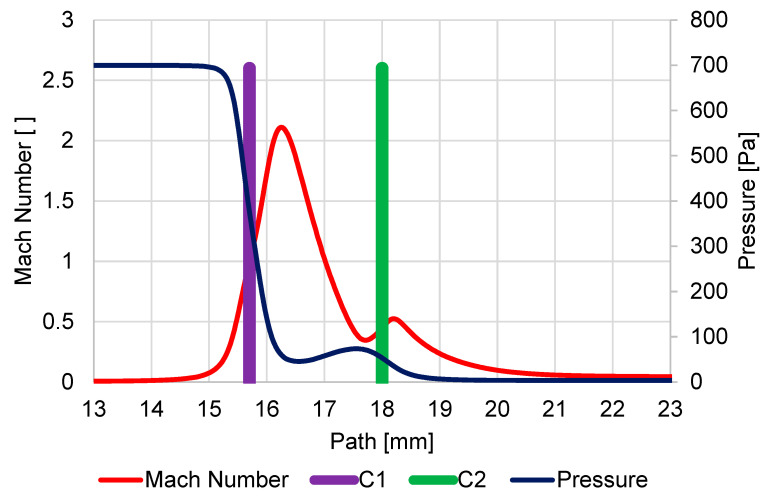
Course of Mach number and pressure on secondary electron path under pressure conditions of 700 Pa in specimen chamber.

**Figure A6:**
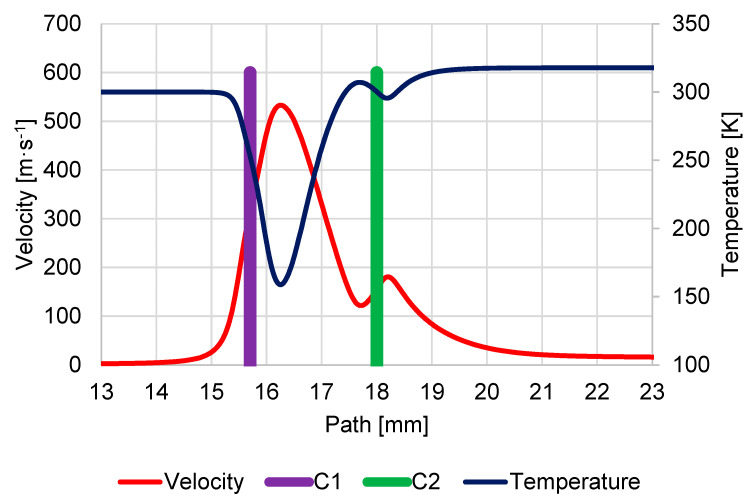
Course of velocity and temperature on secondary electron path under pressure conditions of 700 Pa in specimen chamber.

**Figure A7:**
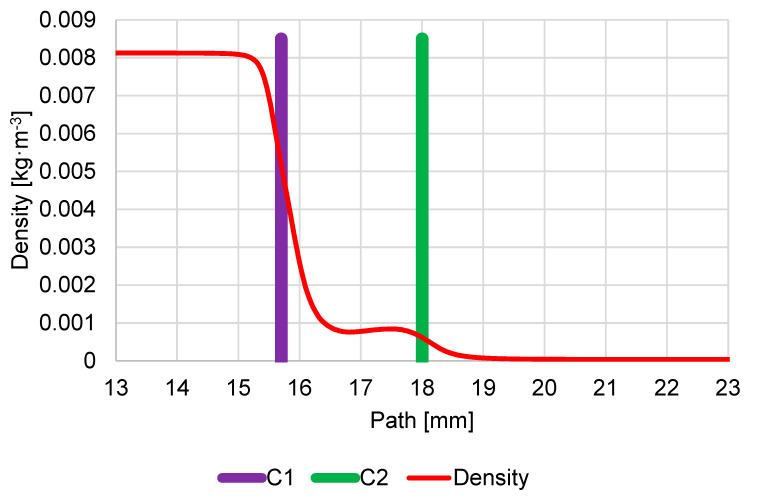
Course of density on secondary electron path under pressure conditions of 700 Pa in specimen chamber.

**Figure A8:**
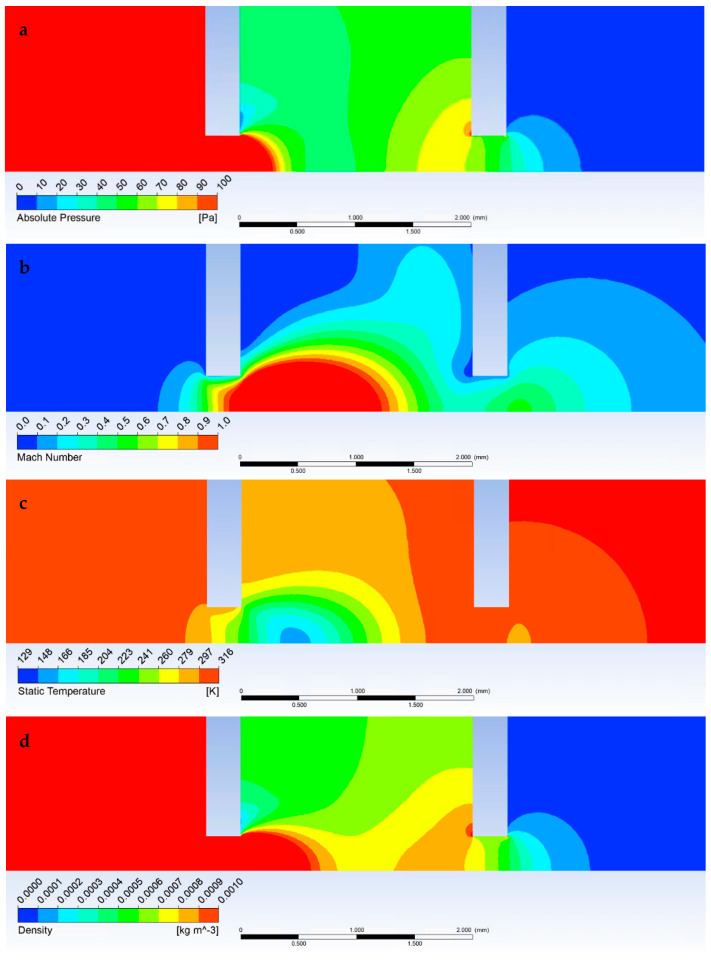
Graphical display of pressure (**a**), Mach number (**b**), temperature (**c**), and density (**d**) of the specimen chamber, pumped inter-chamber and scintillator chamber at 700 Pa pressure conditions in the specimen chamber.

**Figure A9:**
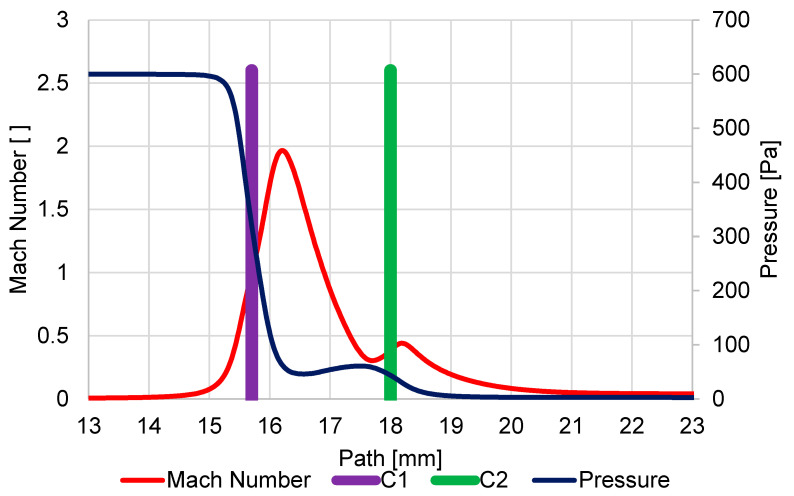
Course of Mach number and pressure on secondary electron path under pressure conditions of 600 Pa in specimen chamber.

**Figure A10:**
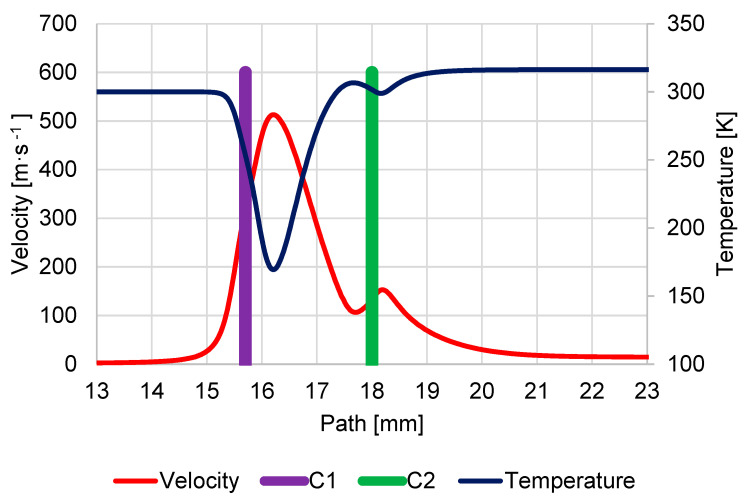
Course of velocity and temperature on secondary electron path under pressure conditions of 600 Pa in specimen chamber.

**Figure A11:**
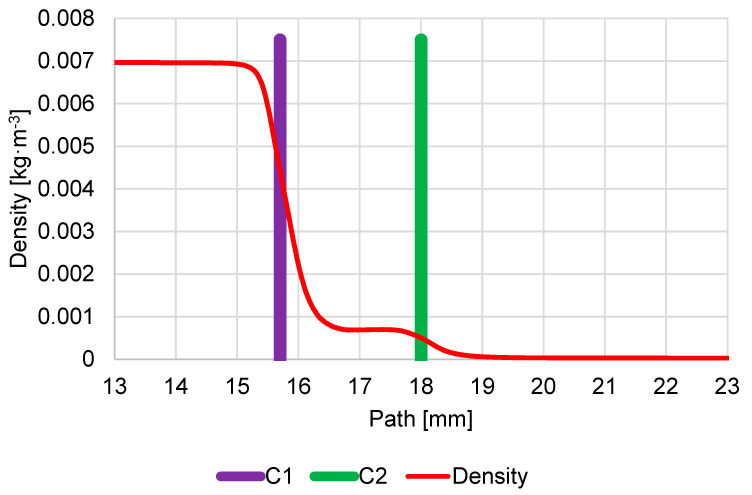
Course of density on secondary electron path under pressure conditions of 600 Pa in specimen chamber.

**Figure A12:**
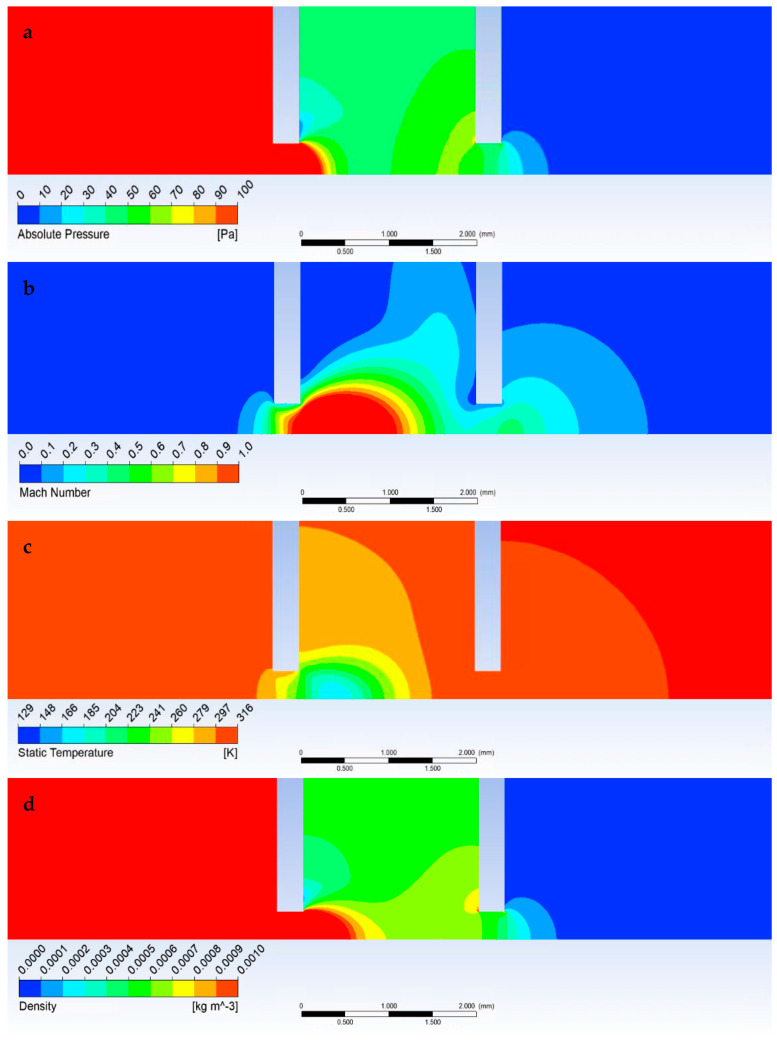
Graphical display of pressure (**a**), Mach number (**b**), temperature (**c**), and density (**d**) of the specimen chamber, pumped inter-chamber and scintillator chamber at 600 Pa pressure conditions in the specimen chamber.

**Figure A13:**
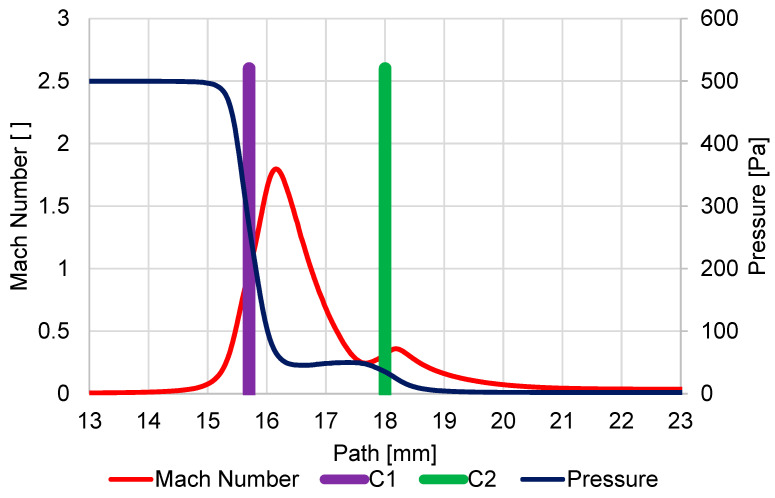
Course of Mach number and pressure on secondary electron path under pressure conditions of 500 Pa in specimen chamber.

**Figure A14:**
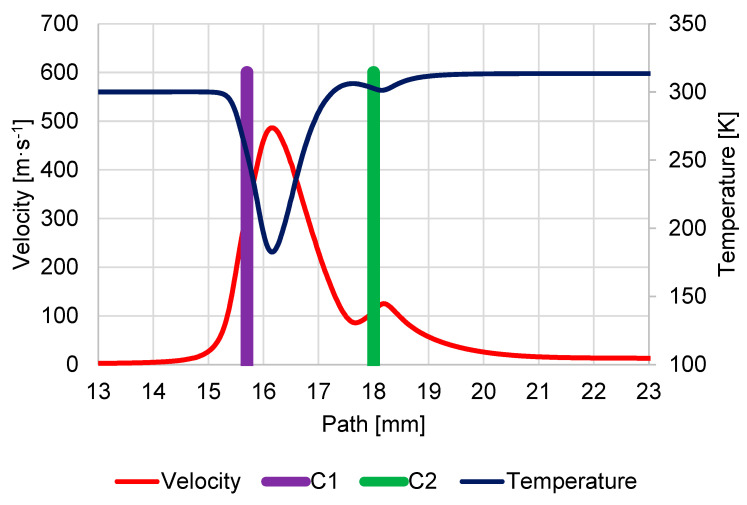
Course of velocity and temperature on secondary electron path under pressure conditions of 500 Pa in specimen chamber.

**Figure A15:**
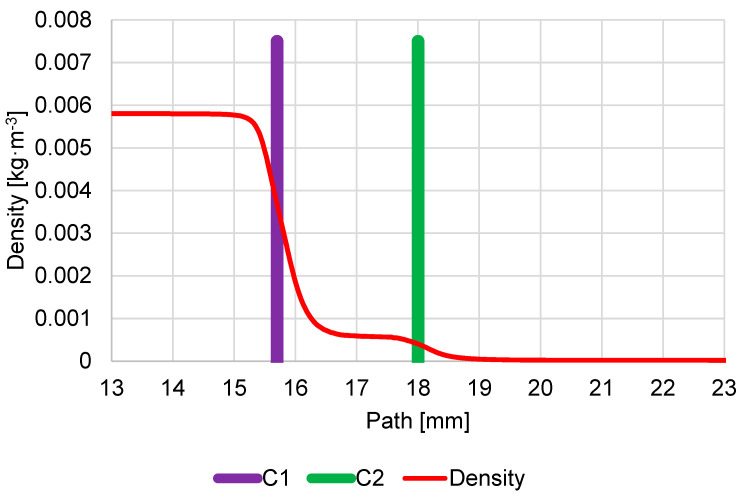
Course of density on secondary electron path under pressure conditions of 500 Pa in specimen chamber.

**Figure A16:**
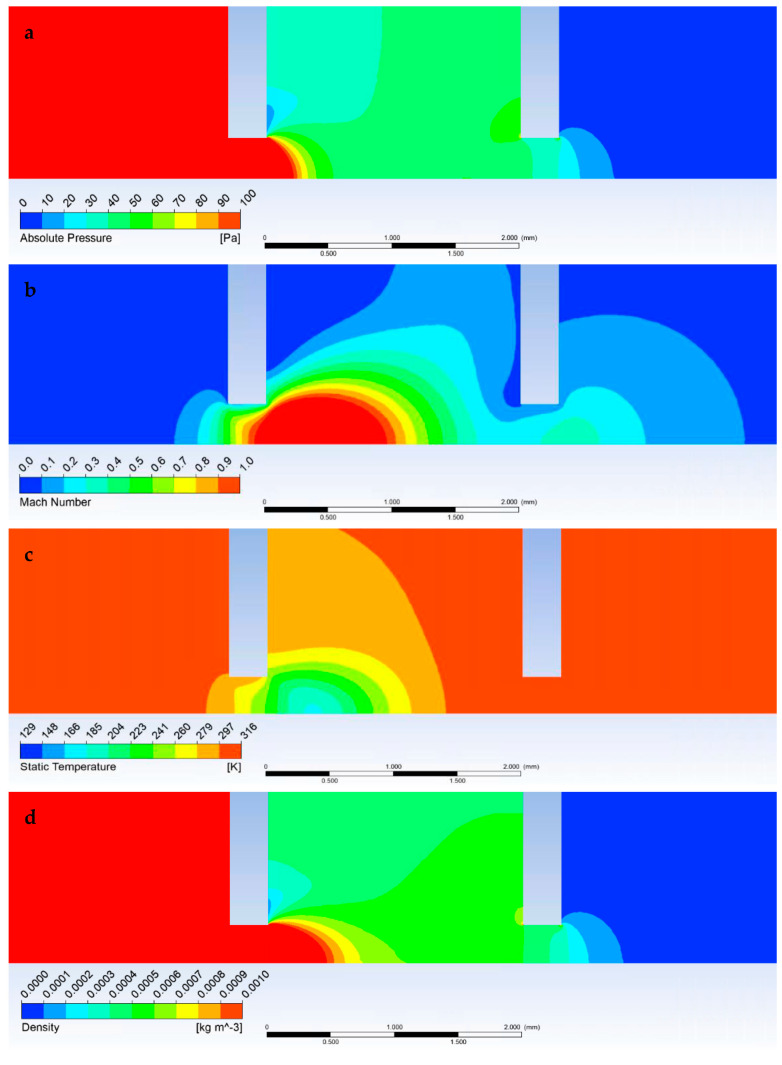
Graphical display of pressure (**a**), Mach number (**b**), temperature (**c**), and density (**d**) of the specimen chamber, pumped inter-chamber, and scintillator chamber at 500 Pa pressure conditions in the specimen chamber.

## Data Availability

The data presented in this study are available on request from the corresponding author.

## Appendix A

### Appendix A.1. Variant 800 Pa in the Specimen Chamber

Here are the courses of Mach number, pressure, flow velocity, temperature, and density on the path of secondary electrons under pressure conditions in the specimen chamber 800 Pa and apertures with the size of the hole diameter C1 = 0.6 mm and C2 = 0.6 mm.

These results also correspond to the above-mentioned theory of one-dimensional isoentropic flow, obtained from Equations (1)–(6), and their comparison is shown in Table A1:

**Table A1:** Comparison of Ansys Fluent results with one-dimensional isoentropic flow theory.

	Ansys Fluent	One-Dimensional Flow Theory
P_0_ [Pa]	800	800
P_v_ [Pa]	75	75
Mach Number [-]	2.34	2.2
Velocity [m·s^−1^]	548	541.9
Temperature [K]	149.6	149.6
Density [kg·m^−3^]	0.001	0.0017
Mach Disk [mm]	1.32	1.31

In the following figures (Figure A4a–d), it is possible to observe the characteristic distribution of the investigated quantities.

### Appendix A.2. Variant 700 Pa in the Specimen Chamber

Here are the courses of Mach number, pressure, flow velocity, temperature, and density on the path of secondary electrons under pressure conditions in the specimen chamber of 700 Pa and apertures with the size of the hole diameter C1 = 0.6 mm and C2 = 0.6 mm.

These results also correspond to the above-mentioned theory of one-dimensional isoentropic flow, obtained from Equations (1)–(6), and their mutual comparison is shown in Table A2:

**Table A2:** Comparison of Ansys Fluent results with one-dimensional isoentropic flow theory.

	Ansys Fluent	One-Dimensional Flow Theory
P_0_ [Pa]	700	700
P_v_ [Pa]	77	77
Mach Number [-]	2.23	2.1
Velocity [m·s^−1^]	532.7	528.9
Temperature [K]	158.2	157.9
Density [kg·m^−3^]	0.0008	0.00169
Mach Disk [mm]	1.24	1.21

In the following figures (Figure A8a–d), it is possible to observe the characteristic distribution of the investigated quantities.

### Appendix A.3. Variant 600 Pa in the Specimen Chamber

Here are the courses of Mach number, pressure, flow velocity, temperature, and density on the path of secondary electrons under pressure conditions in the specimen chamber 600 Pa and apertures with the size of the hole diameter C1 = 0.6 mm and C2 = 0.6 mm.

These results also correspond to the above-mentioned theory of one-dimensional isoentropic flow, obtained from Equations (1)–(6), and their mutual comparison is shown in Table A3:

**Table A3:** Comparison of Ansys Fluent results with one-dimensional isoentropic flow theory.

	Ansys Fluent	One-Dimensional Flow Theory
P_0_ [Pa]	600	600
P_v_ [Pa]	82	82
Mach Number [-]	1.96	1.96
Velocity [m·s^−1^]	532.7	509.3
Temperature [K]	158.2	168
Density [kg·m^−3^]	0.0008	0.00169
Mach Disk [mm]	1.24	1.09

In the following figures (Figure A12a–d), it is possible to observe the characteristic distribution of the investigated quantities.

### Appendix A.4. Variant 500 Pa in the Specimen Chamber

Here are the courses of Mach number, pressure, flow velocity, temperature, and density on the path of secondary electrons under pressure conditions in the specimen chamber of 500 Pa and apertures with the size of the hole diameter C1 = 0.6 mm and C2 = 0.6 mm.

These results also correspond to the above-mentioned theory of one-dimensional isoentropic flow, obtained from Equations (1)–(6), and their mutual comparison is shown in Table A4:

**Table A4:** Comparison of Ansys Fluent results with one-dimensional isoentropic flow theory.

	Ansys Fluent	One-Dimensional Flow Theory
P_0_ [Pa]	500	500
P_v_ [Pa]	87	87
Mach Number [-]	1.8	1.8
Velocity [m·s^−1^]	485.2	484.5
Temperature [K]	182.4	180.3
Density [kg·m^−3^]	0.0008	0.00168
Mach Disk [mm]	1.24	1.14

In the following figures (Figure A16a–d), it is possible to observe the characteristic distribution of the investigated quantities.
